# Clinical applications of and molecular insights from RNA sequencing in a rare disease cohort

**DOI:** 10.1186/s13073-025-01494-w

**Published:** 2025-07-01

**Authors:** Jamie C. Stark, Neta Pipko, Yijing Liang, Anna Szuto, Chung Ting Tsoi, Megan A. Dickson, Kyoko E. Yuki, Huayun Hou, Sydney Scholten, Kenzie Pulsifer, Meryl Acker, Meredith Laver, Harsha Murthy, Olivia M. Moran, Emily Bonnell, Nicole Liang, Jashanpreet Sidhu, Lucie Dupuis, Mohammad M. Ghahramani Seno, Care4Rare Canada Consortium, Marisa Chard, Rebekah K. Jobling, Jessie Cameron, Rose Chami, Michal Inbar-Feigenberg, Michael D. Wilson, David A. Chitayat, Kym M. Boycott, Lianna Kyriakopoulou, Roberto Mendoza-Londono, Christian R. Marshall, James J. Dowling, Gregory Costain, Ashish R. Deshwar

**Affiliations:** 1 Division of Clinical and Metabolic Genetics, The Hospital for Sick Children, Toronto, ON Canada; 2 Program in Developmental and Stem Cell Biology, SickKids Research Institute, Toronto, ON Canada; 3 Program in Genetics and Genome Biology, SickKids Research Institute, Toronto, ON Canada; 4 The Centre for Computational Medicine, SickKids Research Institute, Toronto, ON Canada; 5 Department of Molecular Genetics, University of Toronto, Toronto, ON Canada; 6 Division of Genome Diagnostics, Department of Pediatric Laboratory Medicine, The Hospital for Sick Children, Toronto, ON Canada; 7 Ted Rogers Centre for Heart Research Cardiac Genome Clinic, The Hospital for Sick Children, Toronto, ON Canada; 8 Provincial Medical Genetics Program, NL Health Services, St. Johns, NL Canada; 9 Program in Neurosciences and Mental Health, SickKids Research Institute, Toronto, ON Canada; 10 Discipline of Pediatrics, Faculty of Medicine, Memorial University, St. Johns, NL Canada; 11 Department of Paediatrics, University of Toronto, Toronto, ON Canada; 12 Division of Clinical Biochemistry, Department of Pediatric Laboratory Medicine, The Hospital for Sick Children, Toronto, ON Canada; 13 Department of Laboratory Medicine and Pathobiology, University of Toronto, Toronto, ON Canada; 14 Division of Pathology, Department of Pediatric Laboratory Medicine, The Hospital for Sick Children, Toronto, ON Canada; 15 Prenatal Diagnosis and Medical Genetics Program, Mount Sinai Hospital, Toronto, ON Canada; 16 Department of Obstetrics and Gynecology, University of Toronto, Toronto, ON Canada; 17 Children’s Hospital of Eastern Ontario Research Institute, Ottawa, ON Canada

**Keywords:** RNA sequencing (RNA-seq), Pediatric rare disease, Genetic disease, Molecular diagnostic techniques, Variant of uncertain significance, Putative (new) disease gene, Splice variants, Novel disease mechanisms, Transcriptomics

## Abstract

**Background:**

RNA sequencing (RNA-seq) is emerging as a valuable tool for identifying disease-causing RNA transcript aberrations that cannot be identified by DNA-based testing alone. Previous studies demonstrated some success in utilizing RNA-seq as a first-line test for rare inborn genetic conditions. However, DNA-based testing (increasingly, whole genome sequencing) remains the standard initial testing approach in clinical practice. The indications for RNA-seq after a patient has undergone DNA-based sequencing remain poorly defined, which hinders broad implementation and funding/reimbursement.

**Methods:**

In this study, we identified four specific and familiar clinical scenarios, and investigated in each the diagnostic utility of RNA-seq on clinically accessible tissues: (i) clarifying the impact of putative intronic or exonic splice variants (outside of the canonical splice sites), (ii) evaluating canonical splice site variants in patients with atypical phenotypes, (iii) defining the impact of an intragenic copy number variation on gene expression, and (iv) assessing variants within regulatory elements and genic untranslated regions.

**Results:**

These hypothesis-driven RNA-seq analyses confirmed a molecular diagnosis and pathomechanism for 45% of participants with a candidate variant, provided supportive evidence for a DNA finding for another 21%, and allowed us to exclude a candidate DNA variant for an additional 24%. We generated evidence that supports two novel Mendelian gene-disease associations (caused by variants in *PPP1R2* and *MED14*) and several new disease mechanisms, including the following: (1) a splice isoform switch due to a non-coding variant in *NFU1*, (2) complete allele skew from a transcriptional start site variant in *IDUA*, and (3) evidence of a germline gene fusion of *MAMLD1-BEND2*. In contrast, RNA-seq in individuals with suspected rare inborn genetic conditions and negative whole genome sequencing yielded only a single new potential diagnostic finding.

**Conclusions:**

In summary, RNA-seq had high diagnostic utility as an ancillary test across specific real-world clinical scenarios. The findings also underscore the ability of RNA-seq to reveal novel disease mechanisms relevant to diagnostics and treatment.

**Supplementary Information:**

The online version contains supplementary material available at 10.1186/s13073-025-01494-w.

## Background

RNA sequencing (RNA-seq) is emerging as an important clinical genetic test for the diagnosis of Mendelian disease. RNA-seq enables interrogation of a variant’s effect on RNA transcripts, a capability DNA-testing alone cannot offer. This is particularly useful for variants found in regions of the genome that can be hard to interpret, such as non-coding regions, including untranslated regions and introns. Its diagnostic utility has been demonstrated in multiple rare disease cohorts, including patients with neuromuscular [1] and mitochondrial disease [2, 3], as well as in general cohorts of rare disease patients (Additional file 1: Fig. S1). RNA-seq is increasingly recognized as an important component of the diagnostic toolkit for Mendelian disease [4]; however, its utility and optimal application remain poorly defined.

RNA-seq has demonstrated diagnostic utility in numerous studies, however this has been primarily in the context of an “RNA-first” approach, blinded to any prior or concurrent DNA sequencing results (reviewed in Additional file 2: Table S1) [1–3, 5–12]. While this method has yielded high diagnostic rates, it does not reflect current clinical practices, where DNA testing is almost universally performed first. Consequently, these findings are less pertinent in guiding the practice of clinicians seeking diagnoses in quintessential clinical contexts. Therefore, the indications for RNA-seq following standard clinical genomic testing (e.g., chromosomal microarray analysis, whole exome (WES) or whole genome sequencing (WGS)) remain undefined. Prior transcriptome-first studies have shown variable diagnostic yields (1.5–21%) in patients without candidate variants on exome sequencing [1–3, 5, 6], but the effectiveness of this approach in cases with no candidates on whole genome sequencing (WGS) remains understudied. Addressing these gaps is crucial for the effective integration of RNA-seq into clinical practice.

This study investigates the diagnostic utility of RNA-seq in 53 unrelated individuals with suspected Mendelian disease, using clinically accessible tissues. Specifically, our study was designed to assess the value of RNA-seq in specific instances where it was most likely to be clinically informative, to generate an evidence base to inform implementation and funding/reimbursement. Patients were recruited for RNA-seq if they had previously identified DNA variants meeting one of four specific criteria (described in the Methods below) or had previous short-read whole genome sequencing (WGS) with no candidate variants identified, with a remaining high index of suspicion for an unknown underlying genetic disorder. RNA-seq analysis of the candidate variants (hypothesis-driven RNA-seq) for the first four categories proved highly effective, resulting in a molecular diagnosis for 45% of these 33 probands, improved candidate variant resolution in an additional 21%, and the ability to exclude a candidate variant in another 24%. In addition, the results revealed two putative novel Mendelian disorders and multiple novel molecular mechanisms of disease. Conversely, only a single new putative diagnosis was obtained from RNA-seq in patients with no eligible candidate variant identified on WGS, suggesting limitations to this approach.

## Methods

### Cohort recruitment

**Fig. 1:**
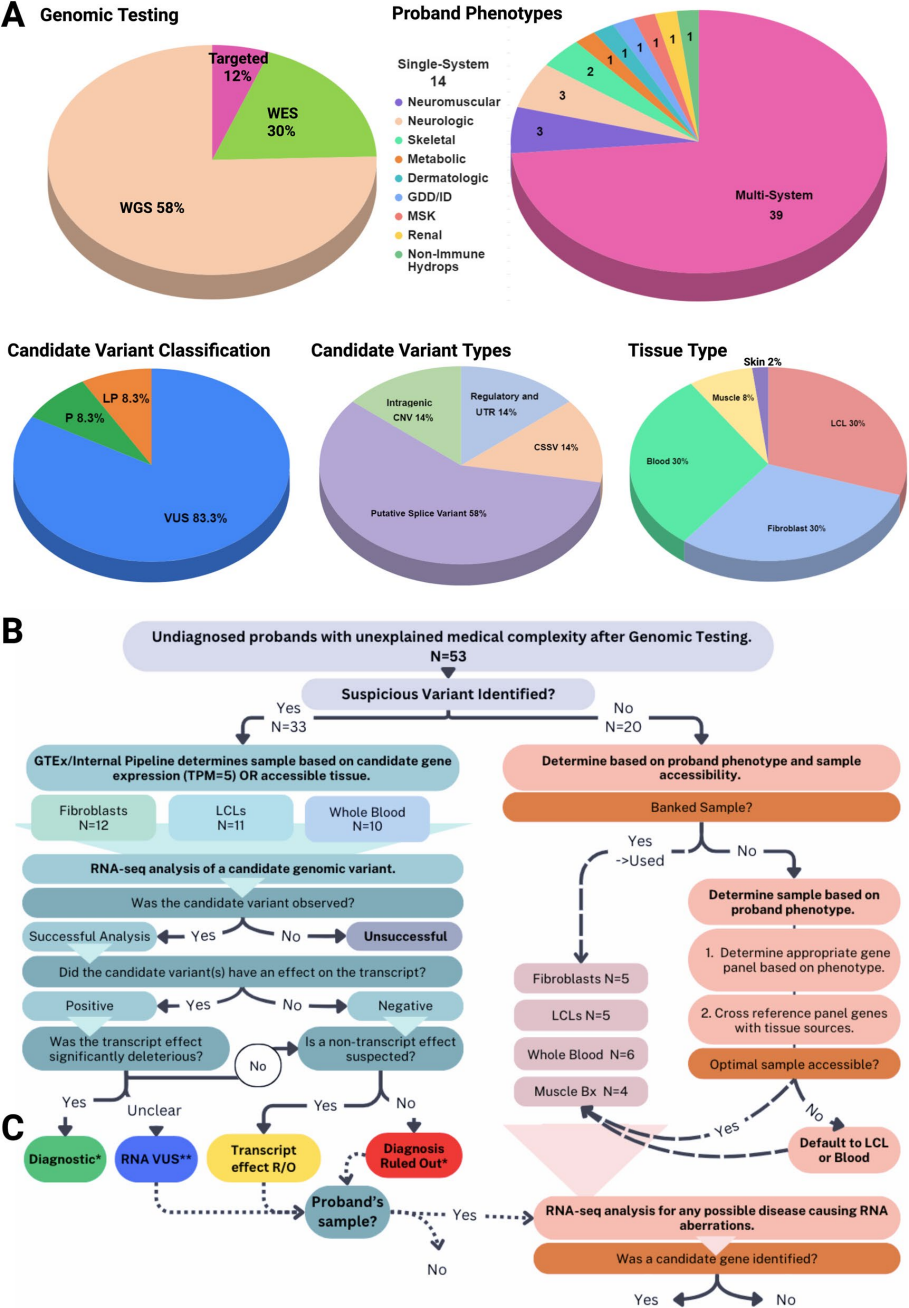
Cohort and study design. **A** Demographics of the rare diseasecohort. Pie charts depict proband phenotypic characteristics, level of prior genomic level testing (targeted only, whole exome sequencing only (WES) and/or whole genome sequencing ± other testing (WGS)), types of variants of interest identified on genomic sequencing, tissue samples used for both RNA-seq, and ACMG classification of candidate variants prior to RNA-seq analysis (P indicates pathogenic, LP indicates likely pathogenic and VUS indicates variant of unknown significance). Phenotypes N numbers are shown, where probands are divided based on multisystem or syndromic presentations characteristics, versus single-system presentations which are further divided based on the involved system. More detailed cohort summaries can be found in Additional file 3: Table S2. Complete phenotype information for each proband can be found in Additional file 4: Table S3. **B** Workflow for selecting tissues for RNA-seq in the presence or absence of a candidate DNA variant. When a candidate variant was identified on genomic sequencing, the GTEx portal [19] was used to determine the appropriate tissue types with baseline expression level of > 5 TPM for the gene of interest. If suitable banked tissue samples were available; they were used for the analysis. The tissue type used for RNA-seq is depicted for each proband based on the gene of interest. RNA-seq analysis focused on the candidate variant was performed initially, and if no pathogenic effect on the transcript was detected, cases were reflexed to hypothesis-independent RNA-seq analysis looking for any putative disease-causing RNA aberrations, when possible. For probands without candidate variants identified, banked tissue was used if available. Otherwise, tissue selection was determined based on expression levels of genes found on typical panels relevant to the proband’s phenotype. If no optimal sample was accessible, LCLs or blood was used. **C** RNA-seq analysis of candidate genomic variant outcome data coding methodology. RNA-seq results successfully enabling analysis of the relevant transcripts were classified as positive (variant affected the transcript) or negative (no difference from controls, meeting ACMG BS3 functional criteria). Positive results, if felt to be significant, were further categorized as “Diagnostic” (impact significantly deleterious, meeting PS3 ± PM1 functional criteria), “RNA VUS” (clinical impact uncertain, not clearly meeting PS3, PM1 or BS3 functional criteria). If a positive result’s, impact was not expected to be clinically significant (meeting BS3 functional criteria), they along with negative results refuted variant pathogenicity from a transcript-effect, and unless another mechanism (i.e., missense) was suspected (these were simply classified as “Transcript effect Ruled Out”), were considered “Diagnosis Ruled Out” cases. RNA-seq data for “Transcript effect Ruled Out,” “Diagnosis Ruled Out” and “RNA VUS” cases underwent analysis for any putative disease-causing RNA aberrations unrelated to the identified candidate, as did the “RNA VUS” cases. *Diagnostic or ruled out outcomes were deemed “diagnostically informative.” **RNA VUSs increased resolution on the candidate DNA variant

Fifty-three probands with suspected genetic conditions were recruited from the Clinical Genetics Clinic at the Hospital for Sick Children (SickKids®; Toronto, Canada) following routine clinical assessment by a Geneticist and after undergoing standard-of-care clinical DNA testing (with or without additional research-based DNA testing) (Fig. 1A, Additional file 3: Table S2). They were referred for consideration of RNA-seq among multiple other possible research tests. No patients were excluded post-referral. The type of RNA-seq analysis conducted (see section below) was determined by previous genetic testing results: either negative WGS with a high residual index of suspicion for an undiagnosed genetic condition, or a candidate DNA variant satisfying one of the four criteria below. Participants were recruited and provided written informed consent under various research studies approved by the Research Ethics Board at the Hospital for Sick Children, including consent for publication of de-identified clinical and research findings [13–18]. Participant’s demographics, phenotype, and clinical genetic testing results were extracted from electronic medical records.

### Determining approach to RNA-seq analysis:

Hypothesis-driven RNA-seq (i.e., informed by a known DNA variant of interest) was performed in four specific clinical scenarios: (i) to clarify the impact of putative intronic or exonic splice variants (outside of the canonical splice sites), (ii) to evaluate canonical splice site variants in patients with atypical phenotypes, (iii) to define the impact of an intragenic copy number variation on gene expression, and (iv) to assess variants within regulatory elements and genic untranslated regions. RNA-seq was also conducted for probands with previous WGS that had identified no candidate variants but where there remained a strong suspicion of an underlying genetic disorder.

### Tissue selection and acquisition for RNA-seq

For patients with a candidate variant, clinically accessible tissues in which the gene is expressed were prioritized. The Genotype-Tissue Expression Portal [19] was utilized to select a tissue where the median TPM (transcripts per million) was greater than or equal to 5. If more than one tissue met this cut-off, an available tissue, such as a previously banked fibroblast line was prioritized. Otherwise, the least invasive tissue was selected (i.e., blood). For probands without a candidate variant, the phenotype and sample accessibility were reviewed to determine the sample tissue to use (Fig. 1B). Clinically accessible tissues used in the study included blood, fibroblasts, lymphoblastoid cell lines (LCLs) and muscle biopsy samples. In five cases, tissue sampling was declined for the proband, and therefore RNA-seq was conducted on a parent or sibling (carrying the variant of interest, if applicable). Fibroblast lines were established from skin biopsies by the Clinical Fibroblast service at the Hospital for Sick Children. LCLs were generated by TCAG (The Centre for Applied Genomics) at the SickKids Research Institute.

### RNA extraction and sequencing

Whole blood was collected in PAXGene Blood RNA tubes (BD Biosciences) and total RNA was extracted using the PAXGene Blood RNA Kit (Qiagen). Fibroblast and Lymphoblastoid cell pellets were extracted using the Qiagen RNeasy Mini Kit. RNA quality and quantity was determined with TapeStation RNA ScreenTape (Agilent). Total RNA was spiked with SIRV Set 3 (Lexogen) by diluting the SIRV stock 1:1000, and then 3.3 µL of the dilution was used for every 100 ng input of RNA for library prep. Libraries were prepared using an automated NEBNext Poly(A) mRNA Magnetic Isolation Module and NEBNext Ultra II Directional RNA Library Prep kit by Illumina (New England Biolabs) on the NGS Workstation (Agilent). Libraries were analyzed for quality using TapeStation DNA High Sensitivity ScreenTape (Agilent) and quantified with KAPA library quantification (Roche) prior to sequencing on a NovaSeq6000 (Illumina) with paired-end 150 bp runs.

### Bioinformatics methods

Reads were first trimmed with fastp (v 0.24.0) [20] and aligned to GRCh38 reference genome with Ensembl [21] annotation (release 104) using STAR (v 2.7.0f) [22] in two-pass mode, and duplicates were marked with Picard (v 2.18.0) [23]. Quality control was done with a variety of tools, including FastQC (v 0.11.5) [24], picard (v 2.18.0) [23], and rnaseqc (v2.3.5) [25] Gene and isoform quantification was performed with RSEM (v1.3.3) [26]. The sashimi plots were generated using ggsashimi (v1.1.5) [27].

Splice junction detection was done using the SJ.out.tab file from STAR [22]. Junctions with at least 5 uniquely mapped reads were considered for further analysis. To represent the usage of a particular junction, a junction score for each of the splice junctions was calculated using the ratio of reads mapping to a particular junction over all the junction reads that had a shared donor or acceptor site. The Z score for each junction was calculated using the GTEx control cohort using the relevant tissue. Aberrant junctions were classified as novel, missing, or outlier based on several criteria: an absolute Z-score ≥ 3, whether the junction was annotated and detected in the GTEx cohort, and whether their usage, as determined by junction score, was increased or decreased. For expression outlier analysis, a Z score was calculated using the sample TPM reported within the GTEx control cohort and genes with an absolute Z score > 2 were considered expression outliers for further analysis. GTEx controls were obtained from GTEx [28] with the following QC metrics filter (RIN > 7, Autolysis less than severe, mapping rate > 80%, intergenic rate < 0.15, rRNA % < 10%) and processed in the same way as our samples. In total, there were 263 whole blood, 288 fibroblast, and 120 LCL samples in the GTEx control cohort. The details of the cutoff scores’ calculation are available at 10.5281/zenodo.15212187 [29].

### RNA-seq analysis and interpretation

For hypothesis-driven RNA-seq analysis in patients with a candidate DNA variant, the gene of interest was examined for expression outlier status and all three types of aberrant splice junctions (novel, missing or outlier) within the region of interest. Each junction was manually reviewed with the Integrative Genomics Viewer (IGV), with comparisons made to at least two tissue-matched internal samples. For duplications, split reads spanning the duplication breakpoints were examined in IGV to confirm whether the duplication event is in tandem. To accurately map and visualize the novel splice junctions created by these split reads in a Sashimi plot, a customized reference genome was created for each case by inserting the duplicated region into the standard reference genome.

For patients without a candidate DNA variant, a phenotype-driven hypothesis-independent RNA-seq approach (HI RNA-seq analysis) was used to filter for genes overlapping with relevant HPO terms. Gene expression analysis was focused on the most significant expression outliers in these genes. Aberrant splice junctions were further filtered based on the patient’s junction score (novel > 0.2; missing/outlier < 0.75) and compared to the corresponding mean junction score in the GTEx [28] reference dataset (novel < 0.2; missing/outlier ≥ 0.6), as observed in at least 80% of the GTEx cohort. Splice junctions showing a larger difference in usage between the patient sample and GTEx were prioritized for further review. Genes with multiple splicing aberrations were also prioritized, as splice-altering variants frequently disrupt more than one junction within a single gene. Splice junctions of interest were manually examined in IGV and compared to tissue-matched internal samples to determine whether they represented true alternative splicing events. When candidate splicing events were identified, available patient genomic data was re-examined to assess if a nearby DNA variant could explain the observed splicing change.

### RNA-seq analysis outcomes

RNA-seq analysis results were categorized into different outcomes, applying current recommendations [30] for interpretation of non-coding variants, based on the American College of Medical Genetics functional (ACMG) criteria where applicable (Fig. 1C). RNA-seq was successful if the transcripts of interest could be analyzed and were classified as positive (variant affected the transcript) or negative (no difference from controls, meeting ACMG BS3 functional criteria) for an observed variant effect on the transcript. If no or only a minor impact was seen on the transcript, then they were classified as”Diagnosis ruled out” except when the variant was felt to possibly directly impact protein function (ie. missense variant) where it was then classified as”Transcript effect ruled out”. RNA-seq data for “Transcript effect Ruled Out,” “Diagnosis Ruled Out” and “RNA VUS” cases underwent analysis for any putative disease-causing RNA aberrations unrelated to the identified candidate.

### IDUA functional studies

A plasmid (pEZX-PF02) containing the IDUA promoter upstream of an eGFP coding sequence was obtained from GeneCopoeia (Catalog# HPRM30186-PF02). A patient variant (NM_000203.5(IDUA):c.−87 T > C) plasmid was generated using the Q5® Site-Directed Mutagenesis Kit (NEB Catalog# E0554S) and verified with Sanger sequencing. HEK-293 cells were plated in a 6-well plate and transfected with the control plasmid or the patient variant construct using Lipofectamine 3000 Transfection Reagent (Thermo Fisher Scientific). Cells were imaged by epifluorescence microscope (Nikon Eclipse Ti2-E; 4 × objective) at 24 h post-transfection. Culture medium was replaced with Dulbecco’s Modified Eagle Medium with 10% fetal bovine serum and 2 µg/mL puromycin for additional 24 h of selection. Cells were then harvested for RT-qPCR targeting *eGFP* and *B2M*. Ct values of *eGFP* were normalized with that of reference gene *B2M* and relative gene expression level was calculated with 2^−ΔΔCt^ method. Unpaired *t*-test was conducted and bar graph with mean ± SEM was plotted with GraphPad Prism 10.3.1 software.

### PDHc activity assay

PDHc activity assay (native and DCA-activated) and PDH subunit enzyme activities were performed as previously described, [31–33] by the SickKids biochemical laboratory.

## Results

### A heterogeneous patient cohort with multisystemic manifestations and suspected Mendelian disease

This study included 53 probands with a suspected Mendelian disease who were all assessed and recruited from the Hospital for Sick Children by a Clinical Geneticist. Seventy-four percent had multisystem involvement (*N* = 39), and 26% (*N* = 14) had single system involvement (Fig. 1A). Detailed phenotypes for each patient can be found in Additional file 3: Table S2. Thirty-three probands were enrolled for investigation of candidate DNA variants under the four scenarios outlined in the methods, 20 individuals had no candidate variants after WGS who underwent RNA-seq (Fig. 1A, Additional file 3. Table S2).

Twenty-nine (88%) of the probands with candidate variants had clinical or research WGS or WES (Fig. 1A, B, Table 1, Additional file 3: Table S2); 86% of these were trios (*N* = 25). In four cases (12%), the candidate variant was identified on microarray, array CGH or an NGS panel. Tissue selection for RNA-seq was performed as described in the methods section (Fig. 1B). For three cases (Case 19, 22 and 28), RNA-seq was conducted on fibroblasts from a parent carrying the variant as a skin biopsy was declined for the proband (9%).

**Table 1:** Summary of the candidate variants with a deleterious impact on the RNA transcript observed by RNA-seq. The first column lists the case ID. The second column details the gene, inheritance pattern of any known Mendelian disease and the transcript of interest NCBI Reference Sequence accession number. The third column details the genomic variant of interest (bolded) as well as in trans variants (not bolded, not under investigation for recessive conditions). It also includes the variant classification (*P* pathogenic, *LP* likely pathogenic, *VUS* variant of uncertain significance), and variant inheritance (maternal (mat), paternal (pat) or de novo). The fourth column presents the findings and outcomes of hypothesis-driven RNA-seq analysis. See Additional file 3: Table S2 for further details including proband phenotypes, and Additional file 1: Fig. S1 for Sashimi plots demonstrating positive RNA-seq findings for cases not highlighted in the main text

Case ID	Gene	Variant(s)	Hypothesis-driven RNA-seq analysis [Outcome]
**Putative splice variant outside canonical splice site**			
**Case 1**	***SASS6*** (AR) NM_194292.3	c.39_49 del (p.Val14 Arg*3)[VUS, mat] **c.207-11 C > A (p.?) [VUS, pat]**	Leaky skipping of exon 4 in 30% of transcripts, causing in-frame deletion including half of the highly considered PISA domain **[Diagnostic]**
**Case 2**	***NFU1*** (AR) NM_ 001002755.4	**c.62 + 89G > A, (p.?) [VUS, mat]** c.545G > A (p.Arg182Gln) [LP, pat]	LP missense variant confirmed to cause skipping of exon 6, with 95% skew to the reference VUS allele. Intronic VUS creates a novel splice donor site, causing exon 1 extension, with a switch towards less biologically relevant transcripts **[Diagnostic]**
**Case 3**	***PPP1R2*** NM_006241.8	**c.403 + 3 A > T (p.?) [VUS, mat and pat]**	Skipping of exon 4 and intron 4–6 retention in most reads, likely triggering a high level of NMD **[RNA VUS]**
**Case 8**	***PIEZO1*** (AD/AR) NM_001142864.4	**c.2991 + 7 C > T [VUS, mat and pat]**	Novel splice donor causing exon 21 extension of 5nt causing frameshift, affecting all reads **[Diagnostic]**
**Case 9**	***ELN*** (AD) NM_000501.4	**c.1719 T > A, p.(V573 =) [VUS, pat]**	Variant causes multiple abnormal splice junctions, likely resulting in NMD, as supported by over 90% skew to the reference allele **[Diagnostic]**
**Case 10**	***FOXRED1*** (AR) NM_ 017547.4	c.733 + 1G > A (exon 6) [P, mat] **c.536 + 5G > A (exon 4) [VUS, pat]**	Both splice donor variants contribute to the skipping of exons 5 and 6 and intron retention in the surrounding region, likely resulting in NMD **[Diagnostic]**
**Case 11**	***PQBP1*** (XLR) NM_ 001032382.2	**c.292 + 5G > A [VUS, mat]**	Variant creates novel splice donor, and exon 4 extension of 12 nucleotides in 22% of transcripts, which likely is in frame. Minor intron retention seen may cause some NMD **[RNA VUS]**
**Case 12**	***PIEZO2*** (AD/AR) NM_022068.4	**c.7743-8 A > G [VUS, mat and pat]**	Variant introduces a novel splice acceptor causing out-of-frame exon 54 extension by 7 nucleotides, seen in all reads. Causes intron 53 retention and loss of exon 53 and 54 expression in 50% of reads **[Diagnostic]**
**Case 51**	***FBXL4*** (AR) NM_001278716.2	c.1303 C > T, p.Arg435* [P, pat] **c.1703-4 A > G [VUS, mat]**	The VUS results in retention of intron 9 in the majority (~ 75–80%) of transcripts, compared to much lower levels seen in tissue-matched control samples. Significant allelic skew (80%) toward the VUS suggests NMD of the transcript carrying the in trans pathogenic variant **[Diagnostic]**
**Case 52**	***ACADM*** (AR) NM_000016.6	c.984 del; p.Met328Ilefs*5 [P, mat**]** **c.85 C > A; p.Arg29 = [VUS, pat]**	The synonymous variant significantly increased skipping of exon 2 in ~ 50% of reads, compared to minimal seen in tissue-matched controls, resulting in a frameshift **[Diagnostic]**
**Case 53**	***UFC1*** (AR) NM_016406.4	**c.333-14 T > C p.? [VUS, mat]** c.435G > A p.Trp145* [VUS, pat]	The splice acceptor variant induces a tenfold increase in skipping of exon 5, an alternative exon, leading to a frameshift and truncation at the C-terminus. This suggests a switch towards a less biologically relevant transcript **[RNA VUS]**
**Canonical splice site variant (CSSV)**			
**Case 13**	***EFTUD2*** (AD) NM_004247.3	**c.702 + 1 del (p.?) [P, de novo]**	Novel splice donor causes shortening of exon 10 by 1 nucleotide in 11/89 reads causing a frameshift and decreased mRNA expression **[Diagnostic]**
**Case 14**	***TBX6*** (AR/AD) NM_004608.4	**c.118 + 2 T > C [P, pat]** T-C-A haplotype (c.1227G > A, c.−48-240 A > G, −49 + 34G > T) [risk allele, mat]	Paternal splice donor variant causes exon skipping of first exon leading to N-terminal truncation, seen in 80% of the proband's transcripts (compared to 30% in the father) **[RNA VUS]**
**Case 15**	***MED14*** NM_004229.4	**c.2365 + 2 T > C [VUS, mat]**	Use of novel splice donor causes out-of-frame loss of the C-terminal end of exon 18 in 1.7% of transcripts **[RNA VUS]**
**Case 16**	***RABGAP1*** NM_012197.4	**c.591-1G > T [VUS, mat and pat]**	Splice acceptor variant causes 8 bp loss from 5'end of exon 5 resulting in frameshift in most reads, while a few demonstrate intron retention predicted to cause frameshift. There are no reads with normal splicing **[RNA VUS]**
**Case 17**	***SYNGAP1*** (AD) NM_ 006772.3	**c.1914-1G > C [P, inheritance unknown; not mat]**	Variant results in creation of novel splice acceptor site within exon 12, causing 13 bp shortening of exon 12 which is out-of-frame. Skew is 77% toward the reference allele. Also, low levels of retention of surrounding introns **[Diagnostic]**
**Compound heterozygote for putative splice variant and CNV**			
**Case 18**	***TGM1*** (AR) NM_000359.2	**c.985-3 C > G, [VUS, de novo]** ** ~ 3.58 kb Duplication** **Chr14:24,253,090–24256672 (GRCh38), [LP, mat]**	Duplication in-tandem and out-of-frame with intron retention. Splice acceptor variant causes out-of-frame exon 7 skipping in 50% of transcripts **[Diagnostic]**
**Structural variant (SV)/intragenic copy number variant (CNV)**			
**Case 19**	***COL4 A5*** (XLD)	** ~ 23 kb duplication; exons 10–24** **chrX:108,574,886–108,598,389 (GRCh38) [LP, mat]**	Duplication is in tandem and in frame **[Diagnostic]**
**Case 20**	***CDKL5*** (XLD)	**105 kb duplication; exons 2–5** **ChrX:18,474,185–18,579,246 (GRCh38) [LP, presumed de novo]**	Duplication is in-frame in-tandem, causing some likely out-of-frame intron retention **[Diagnostic]**
**Case 21**	***TONSL*** (AR) NM_013432.5	c.1459G > A:p.Glu487Lys [P, pat] **467 bp exonic duplication; exon 25** **Chr8: 144,430,363–144430830** **(GRCh38) [VUS, mat]**	Duplication is in-tandem and out-of-frame. No duplication seen in proband’s sample **[Diagnostic]**
**Regulatory or untranslated region variants**			
**Case 24**	***IDUA*** (AR) NM_000203.5	c.1205 G > A, p.Trp402 Ter [P, pat] **c.−87 T > C [VUS, mat]**	VUS results in 97% skew toward the reference pathogenic allele **[Diagnostic]**
**Case 28**	***MAMLD1*** (XLR)	** ~ 407 kb duplication** **ChrX:149,960,910–150367537 (GRCh38) [VUS, mat]**	Duplication not in-tandem. Split reads map from 3'end of duplication to exons 7 and 8 of a distant X chromosome gene *BEND2*, suggesting a fusion event and partial expression of *BEND2*, which is absent in tissue matched controls **[RNA VUS]**
**Hypothesis-Independent RNA-Seq analysis in patients without a DNA candidate variant**			
**Case 44**	***HUWE1*** (XLR) NM_031407.7	**c.10035 + 21 T > A [VUS, mat]**	Leaky out-of-frame skipping of exon 67 observed in 26% of transcripts, predicted to cause a frameshift. Associated variant identified upon re-evaluation of WGS data **[RNA VUS]**

### RNA-seq clarifies the impact of putative splicing variants outside of the canonical splice sites and reveals novel disease mechanisms

**Fig. 2:**
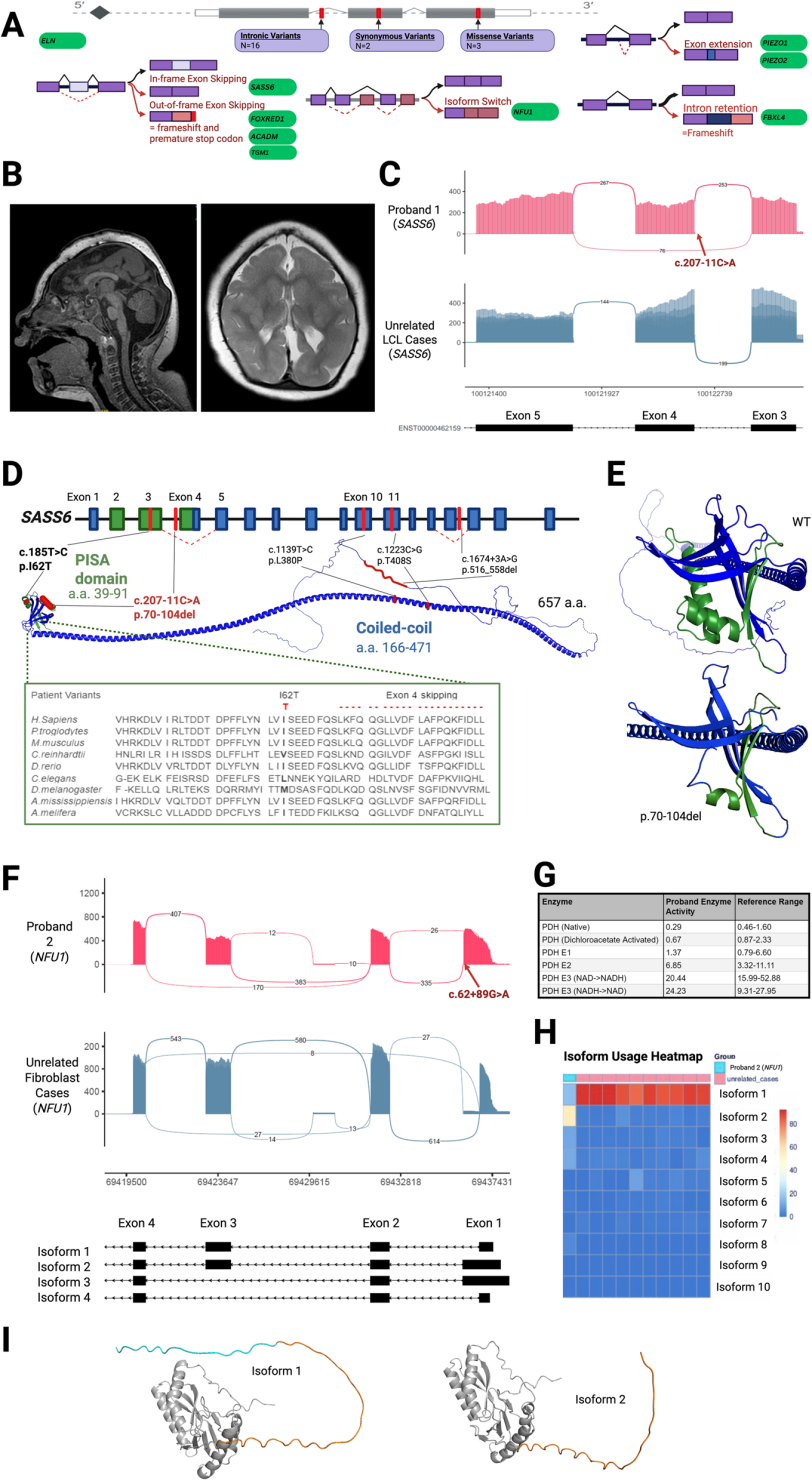
RNA-seq results for probands with candidate putative splicing variants. **A** Summary of positive RNA-seq results for putative splicing variants (depicted in red). Two candidate variants were synonymous, three were missense and fifteen were intronic. Diagnostic variant effects causing abnormal splicing are demonstrated in red, compared to normal splicing in black. In-frame exon skipping was seen in Case 1, out-of-frame exon skipping causing frameshift and premature stop codon was seen in Cases 10 and 52. Isoform switching to less biologically relevant transcripts was seen in Case 2. Exon extension was seen in Cases 8 and 12 and intron retention was seen in Case 51. In Case 9, several disrupted splice junctions were observed near the variant, causing near-complete allele-skew likely due to nonsense-mediated decay. **B** Case 1: Proband 1 Sagittal T1 and Axial T2 MRI Brain demonstrating severe microcephaly with abnormality of neuronal migration/organization, dysmorphic corpus callosum and brainstem, hypoplastic left olfactory bulb and right probable persistent hypertrophic primary vitreous and incidental pituitary cyst. **C** Case 1: Sashimi plot of *SASS6* RNA-seq data in LCLs demonstrating mild in-frame skipping of exon 4 in some transcripts (superior, red) caused by the c.207-11 C > A *SASS6* variant in Proband 1, in contrast to canonical splicing seen in unrelated LCL cases (inferior, blue). **D** Case 1: *SASS6* exon 4 skipping results in partial deletion of the highly conserved PISA domain. Proband 1’s variants, as well as previously reported disease-causing non-truncating variants in *SASS6* are also demonstrated (in red). One is a missense variant (c.185 T > C, bolded) encoding a residue within the PISA domain which is highly conserved across multiple species. **E** Case 1: Superior image shows reference wildtype SASS6 protein model generated by ColabFold [34], demonstrating normal SASS6 folding. The PISA functional domain is depicted in purple. Inferior image shows model of proband 1’s SASS6 protein folding, as predicted by ColabFold, demonstrating the impact of SASS6 p.70-104 del including part of the PISA functional domain (purple) resulting from exon 4 skipping induced by Proband 1’s c.207-11 C > A variant. **F** Case 2: Sashimi plot of *NFU1* RNA-seq data in fibroblasts demonstrating extension of exon 1 and reduced coverage of exon 3, linked to the c.62 + 89G > A variant in Proband 2 (red), consistent with alternative isoforms (2, 3, and 4 shown in black below the plot). In contrast, normal splicing patterns seen in controls (blue). The predominant *NFU1* isoforms are displayed in the inferior panel in black. **G** Case 2: *NFU1* isoform usage in proband 2 compared to tissue-matched samples. The heatmap demonstrates RSEM (RNA-Seq by Expectation-Maximization) isoform percentages from 0% (blue to 100% (red). Proband 2’s isoform usage is shown in the first column, and tissue matched samples are in the following right-hand columns. NFU1 isoform usage in proband 2 is markedly skewed away from isoform 1 (ENTS00000410022, 15%) and toward isoforms 2 (ENTS00000303698, 56%), 3 (ENTS00000394305 at 10%) and 4 (ENTS00000450796 at 11%) compared to controls. **H** Case 2: Superior image shows reference predominant NFU1 isoform 1 (ENTS00000410022) protein model generated by ColabFold [34], where the first coding exon sequence is depicted in blue and the second exon is in orange. Inferior image shows model of NFU1 isoform 2 (ENTS00000303698) as generated by ColabFold, predominant in proband 2 due to the c.62 + 89G > A variant. Isoform 2 uses a more downstream start codon resulting in N-terminal shortening compared to isoform 1, with loss of the amino acids from the first coding exon as well as the second exons first three amino acids within the protein. **I** Case 2: Proband 2’s pyruvate dehydrogenase (PDH) enzyme activity is charted in comparison to reference ranges, demonstrating low PDH enzyme activity (both native and DCA-activated), normal PDH subunit enzyme testing (E1, E2 and E3), and an elevated Lactate/Pyruvate ratio. **J** Case 3: Clinical photographs of proband 3 taken at ages 6 (top) and 11 (bottom) demonstrating distinctive facial features

Nineteen of the 53 probands had putative splicing variants outside the canonical splice sites in genes felt to possibly explain their features. In these individuals, 21 unique variants of uncertain significance (VUS) were interrogated through RNA-seq (2 probands had compound heterozygous putative splice variants, and note that Case 18 is a compound heterozygote with an intragenic CNV that is also included in an upcoming section). Among these variants, 16 were intronic, 2 were synonymous, and 3 were missense variants with predicted possible splicing effects (see Fig. 2A). RNA-seq was successful in confirming or refuting a transcript effect for all 21 variants across 19 probands (100%). Thirteen variants across 13 probands (68%) were positive for a splicing impact, which were considered diagnostic in nine of the probands (47%) (Fig. 2A). Exon skipping events were seen in five of these diagnostic cases; in Case 1 (highlighted below), it resulted in in-frame loss of part of a highly conserved domain; in Cases 10, 18, and 52, it resulted in an out-of-frame transcript and frameshift; and in Case 2 (highlighted below), it resulted in isoform switch to less biologically relevant transcripts. Exon extension resulting from partial intron retention was seen in two diagnostic cases where it caused a frameshift (Case 8 and 12). Intron retention was seen in another diagnostic case (Case 51). In Case 9, multiple abnormal splice junctions and skewed allelic expression were observed, suggesting nonsense-mediated decay (NMD) of the abnormal transcripts, which was diagnostic.

For three probands (Case 3, 11 and 53) with five unique variants, transcript effects were seen, but their clinical significance was considered uncertain. In Case 3, highlighted below, exon-skipping was observed to cause an out-of-frame transcript, as well as intron retention, but given the variant is in a putative disease gene, the diagnosis is not definitive. In Case 11, in-frame exon extension was observed in only ~ 22% of transcripts. In Case 53, increased use of an alternate exon was seen causing a frameshift at the C-terminal end of the protein making its significance unclear. In Case 4, a compound heterozygote for two candidate variants in a gene associated with recessive disease, a mild splice effect was observed for only one of the two variants, ruling out the diagnosis for the proband. RNA-seq was negative for seven other variants across six probands (Cases 5, 6, 7, 23, 49, 50), as no impact on splicing was seen. Pathogenicity from a protein standpoint could not be ruled out for proband 23 and 50 since they were missense variants. For the seven probands with negative RNA-seq and the three with uncertain results (Case 3, 11, 53), RNA-seq data was assessed for alternate candidates (results described below). Findings for all cases can be found in Table 1 and Additional file 4: Table S3. Further details regarding notable cases demonstrating the utility of RNA-seq in this molecular context are provided below.

### Case 1—*SASS6*

Proband 1 had a history of congenital microcephaly, global developmental delay, seizures, and exudative vitreal retinopathy. Brain MRI revealed abnormal neuronal migration, dysmorphic corpus callosum and brainstem, hypoplastic left olfactory bulb, right probable persistent hypertrophic primary vitreous, and a pituitary cyst (Fig. 2B). Trio-ES identified novel compound heterozygous variants including a frameshift c.39_49 del p.(Val14 Arg*3) variant and an intronic c.207-11 C > A VUS in *SASS6* (NM_194292.3). Since the gene’s initial association with autosomal recessive (AR) primary microcephaly in 2014, seven causative *SASS6* variants (all missense or putative splice variants) have been reported in seven individuals across four families [35–38]. For the c.207-11 C > A intronic variant, there was no consensus between four in silico splicing prediction tools (SSF, no change; MaxEnt, − 16.3% change; NNSPLICE, − 0.4% change; GeneSplicer, 100% change). RNA-seq on the proband’s LCLs to investigate the intronic VUS revealed that the c.207-11 C > A variant led to in-frame skipping of exon 4 in approximately 1/3 of all transcripts (Fig. 2C) and consequently loss of the majority of a highly conserved PISA domain (Fig. 2D and E), essential for SASS-5 interaction [35, 39]. This result suggests a likely hypomorphic impact on the protein and supports that total loss of SASS6 function is likely lethal.

### Case 2—*NFU1*

Proband 2 had a history of GDD, seizures, upper limb hypertonia, low central tone with hyperreflexia and clonus, and motor regression. He had multiple ICU admissions for decline of neurological status after a viral illness and died at 3 years of age from acute neuromuscular respiratory failure. Mitochondrial DNA sequencing was negative. Trio-WGS identified the c.545G > A likely pathogenic missense variant in trans with an intronic c.62 + 89G > A VUS in *NFU1* (NM_001002755.4). Biallelic *NFU1* variants are associated with multiple mitochondrial dysfunction syndrome, a rare condition characterized by white matter lesions, neurologic regression, and several biochemical abnormalities (elevated lactate, decreased pyruvate dehydrogenase (PDH) complex activity, decreased respiratory chain complex I and II activity among others). While proband 2 experienced some features consistent with this condition, including neurological regression that worsened with illness and a low total pyruvate dehydrogenase (PDH) activity (Fig. 2G), he had a largely unremarkable brain MRI with spectroscopy, normal blood and CSF lactate levels, and normal mitochondrial complex activities in both muscle and skin biopsies. RNA-seq of the proband’s fibroblasts showed that the c.545G > A variant resulted in out-of-frame skipping of exon 6, and in a minority of reads skipping of exon 6 and 7 as previously described [40]. Additionally, there was almost complete allele skew away from the likely pathogenic allele, indicating NMD. This is supported by further findings [40], which demonstrate the absence of mature protein from this transcript. On the other hand, the c.62 + 89G > A variant resulted in the extension of exon 1 (Fig. 2F). Interestingly, this extended exon corresponds to an alternate transcript (isoform 2, Fig. 2F, H) which relies on a downstream start codon to preserve the reading frame, resulting in N-terminal truncation of the protein (F ig. 2I). There was additionally reduced coverage of exon 4, corresponding to two other splice isoforms (isoforms 3 and 4, Fig. 2F). Altogether, this suggests an isoform switch from the normal predominant transcript to three alternate transcripts which are typically expressed at very low levels (Fig. 2H), providing a putative explanation for his atypical presentation.

### Case 3—*PPP1R2*

Proband 3 had a history of pre and postnatal growth restriction, ventricular septal defect, dysmorphic features (proptosis, long eye lashes, thick eyebrows, low-set ears), microcephaly, sensorineural hearing loss, cortical cataracts, retinal defects, intellectual disability with limited speech, and autism spectrum disorder (ASD). The parents are consanguineous. Trio-WES identified a homozygous c.403 + 3 A > T VUS in a candidate gene, *PPP1R2* (NM:006241.8); the variant was absent in an unaffected brother. Pathogenic variants in *PPP1R2* have not been reported in association with human disease; however, *PPP1R2* is ubiquitously expressed and is known to play a role in cell division, tubulin acetylation, neuronal cell survival, and cardiac function [41–43]. *PPP1R2* knockout mice are embryonically lethal, while heterozygotes have no observable phenotype [44]. *PPP1R2* additionally interacts with other protein phosphatases which have been associated with syndromic neurodevelopmental disorder with many overlapping features with the proband [45–47]. In silico prediction tools unanimously indicate the complete abolishment of splicing at the canonical donor site of exon 4, which is compelling considering that complete loss of function is embryonically lethal. RNA-seq in whole blood revealed skipping of exon 4 in a significant proportion (~ 80%) of reads (Additional file 1: Fig. S1 A) along with a low level of intron 4–6 retention. While splicing was disrupted in a high proportion of reads, a fraction of normally spliced transcripts was also observed, which may explain the survival of the proband.

### RNA-seq in probands with canonical splice site variants and atypical phenotypes provides insight into the molecular mechanisms of disease

**Fig. 3:**
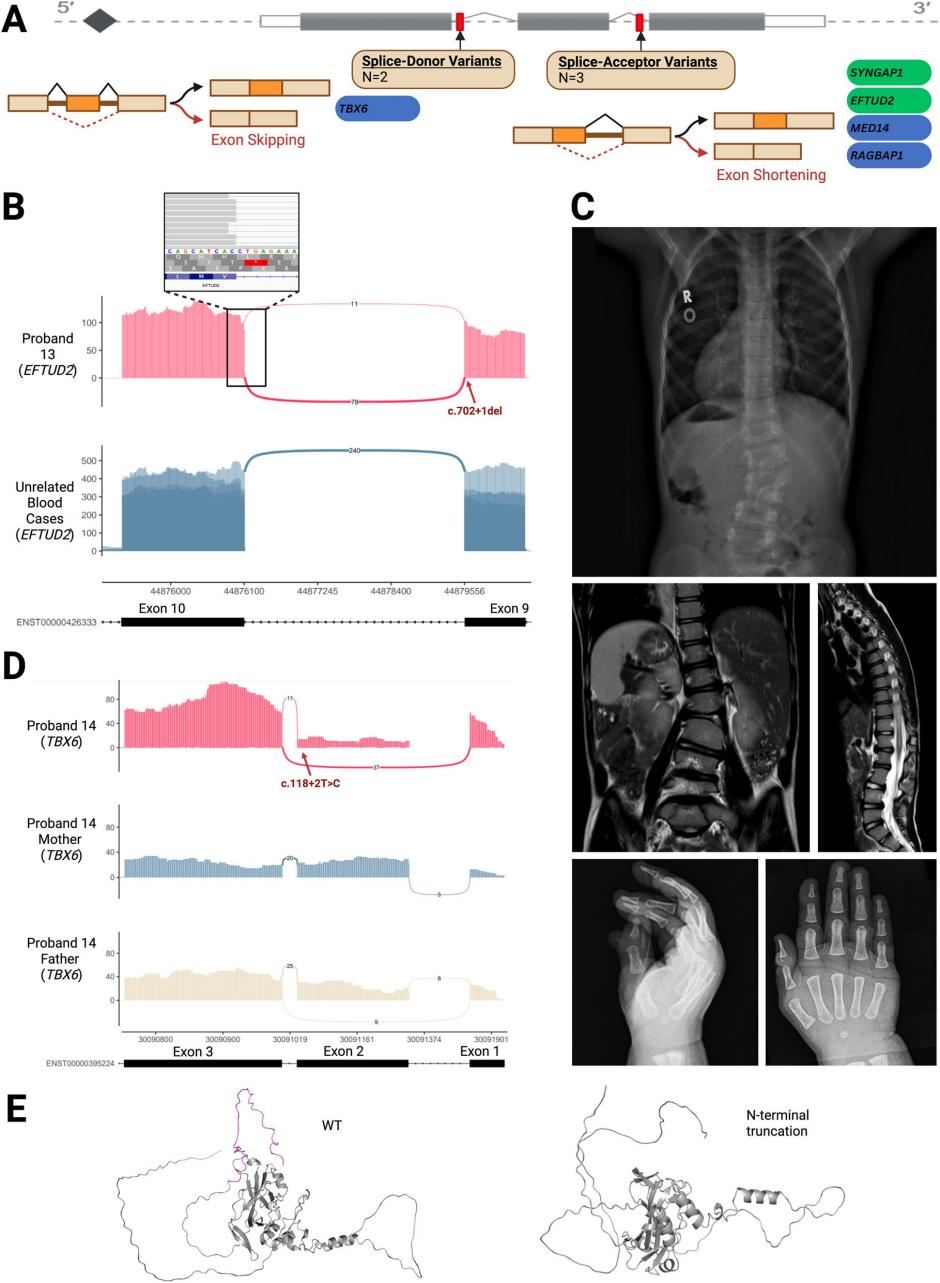
RNA-seq Results for Probands with Canonical Splicing Variants and Atypical Phenotypes. **A** Summary of positive RNA-seq results for CSSVs. 2 variants under investigation (depicted in red) were at canonical donor splice sites and 3 were at acceptor sites. 4 variants lead to out-of-frame exon-shortening causing frameshift (Cases 13, 15, and 16, left) and 1 variant caused out-of-frame exon skipping causing frameshift (Cases 14 and 17). **B** Case 13: *EFTUD2* Sashimi plots display out-of-frame splicing in Proband 13 at the variant site compared to tissue-matched samples. The superior panel displays the proband’s blood RNA-seq results at the *EFTUD2* locus (red) which showcases out-of-frame splicing in 11 reads resulting from the c.702 + 1 delG variant, compared to unrelated blood RNA-seq cases where all reads display normal splicing (blue). The superior call-out displays the Integrative Genomics Viewer graphics at the splice site, demonstrating that the abnormal splicing causes a 1 bp shorter transcript in ~ 14% of reads, predicted to result in a frameshift. **C** Case 14: Proband 14 X ray and MRI demonstrate situs inversus and polydactyly. The top panel is a chest x-ray demonstrating situs inversus totalis, hypoplastic right third rib, left L2 hemivertebrae and levoconvex scoliosis of 26 degrees. Middle panels show MRI spine demonstrating abdominal situs inversus, levoconvex curvature deformity with left hemi vertebra at L2. Middle right panel shows segmental expansile syrinx at T8 to the conus with smaller segmental expansion at C5-T1. There is also mild distention of the central canal in the thoracic cord. Inferior panels are right hand X-rays demonstrating right pre-axial polydactyly. **D** Case 14: *TBX6 s*ashimi plots from Proband 14 at the variant site compared to unaffected parents. Skipping of exon 2 caused by the c.118 + 2 T > C variant is seen in 80% of proband 14’s transcripts (superior, red), while the same event is seen in 30% of transcripts in his father (middle, yellow). Skipping is not seen in his mother as she does not carry the splice variant (bottom, red). **E** Case 14: Superior image shows reference wildtype TBX6 protein model generated by ColabFold, [34] demonstrating normal TBX6 folding. The N-terminal domain is depicted in purple. Inferior image shows model of proband 14’s TBX6 protein folding, as predicted by ColabFold, demonstrating the impact of N-terminal truncation of TBX6 due to use of the M53 downstream start codon, as predicted to result from exon 2 skipping induced by Proband 14’s c.118 + 2 T > C variant

Five unique canonical splice site variants (CSSVs) in five patients were investigated with RNA-seq in probands possessing variants in a known disease gene presenting with an atypical phenotype (three probands), or where a CSSV was seen in a putative new disease gene (two probands). While all three variants in known disease genes were classified as pathogenic, the atypical phenotype seen in the patient raised uncertainty as to the explanation for their unique features. New candidate genes with CSSVs were included given a recent study [48] found that 25% of rare CSSVs do not cause loss-of-function, increasing the need for confirmation of CSSVs before drawing a new disease association. Three variants were in canonical splice donor sites (+ 1 or + 2), while two were in canonical splice acceptor sites (− 1 or − 2) (Fig. 3A). RNA-seq was successful for all five variants (100%), and all were positive (i.e. were confirmed to affect splicing) (Fig. 3A). In every case, RNA-seq helped elucidate a molecular mechanism, providing a compelling possible explanation for the unique phenotype and/or revealing the molecular mechanism of a potential novel disease. Three results were considered diagnostic for the proband; one case demonstrated out-of-frame exon shortening (Case 13) but only in minority of reads explaining a mild phenotype, another case of out-of-frame exon shortening (Case 17), and one case was in a new disease gene where additional patients were identified confirming its association with disease (Case 16, previously published [49, 50]). Further evidence is still required to solidify a diagnosis for the other two cases, both highlighted below which include a putative new disease gene (Case 15) with a mild impact on splicing and a novel transcript encoding a truncated protein (Case 14). For both uncertain cases, RNA-seq data was also assessed for alternate candidates. Findings are summarized in Table 1 and detailed in Additional file 4: Table S3. Specific outcomes demonstrating the utility of RNA-seq in this molecular context are highlighted below.

### Case 13—*EFTUD2*

Proband 13 had a history of tracheoesophageal fistula, unilateral microtia, bilateral hearing loss, bilateral Mondini malformation, and scoliosis. Trio-WES identified a heterozygous de novo c.702 + 1 delG variant in *EFTUD2* (NM_004247.3), interpreted as pathogenic based on ACMG guidelines. Heterozygous loss-of-function variants in *EFTUD2* are associated with mandibulofacial dysostosis with microcephaly (MIM#610,536); however, this 23-year-old female never had microcephaly, cognitive impairment, or mandibulofacial dysostosis on examination. Whole blood RNA-seq was performed to determine whether an unanticipated splicing effect from this variant may explain the atypical phenotype. The splice donor variant created a novel donor site in exon 10, resulting in a 1 bp frameshift. However, this only occurred in a minority (11/89, 12%) of reads (Fig. 3B), and the total number of reads was not reduced compared to tissue-matched control samples. This reduced impact on the transcript is consistent with the milder phenotype observed in the proband.

### Case 14—*TBX6*

Proband 14 had a history of L3 hemivertebra with congenital thoracolumbar scoliosis, segmental expansile syrinx, pre-axial polydactyly, and situs inversus totalis with dextrocardia (Fig. 3C). Trio-WGS identified a splice site c.118 + 2 T > C variant (paternally inherited) reported as pathogenic, *in trans* with the well-documented permissive T-C-A haplotype (maternally inherited), in the *TBX6* gene (NM_004608.4). The T-C-A haplotype, when in trans with a loss-of-function *TBX6* variant, is associated with congenital scoliosis and more recently kidney malformations [51]. However, polydactyly and situs inversus have not been associated with this condition, although interestingly heterotaxy has been observed in *TBX6* knockout mice embryos [52]. RNA-seq of LCL’s in the proband revealed that the + 2 variant results in the skipping of exon 2, which is the first coding exon of the gene (Fig. 3D). A downstream start codon can be found in exon 3, suggesting that this variant generates an N-terminally truncated protein (Fig. 3E). This skipping event was found in most transcripts (80%) in the proband compared to his unaffected father (30%), consistent with the decreased expression of the alternate allele due to the maternally inherited permissive haplotype (Fig. 3D). The predominance of the novel truncated TBX6 transcript provides a putative mechanism for the atypical phenotype seen in this proband but requires further study.

### Case 15—*MED14*

Proband 15 presented with developmental delay, microcephaly, and hypotonia. Trio-WGS revealed a canonical splice site VUS in a candidate gene, *MED14* (NM_004229.4), c.2365 + 2 T > C, as well as a diagnosis of VLCAD that was not felt to contribute to his phenotype. *MED14* encodes a protein forming part of the Mediator Complex, which is essential for gene expression. *MED14* is an X-linked gene highly intolerant to loss of function, with zero individuals with hemizygous loss of function alleles in gnomAD [53, 54] (pLI: 1). Therefore, loss of function in males is expected to be lethal and its role as an essential gene is also supported in model organisms [54]. RNA-seq of LCLs revealed that the variant lead to out-of-frame exon 18 shortening in a minority (~ 1.7%) of transcripts (Additional file 1: Fig. S1B). Collectively, these findings offer a molecular explanation for the presence of a canonical splice site variant in a viable male and suggest the possibility of a novel Mendelian disorder arising from a slight reduction in MED14 function, however the clinical significance of this event remains unconfirmed.

### RNA-sequencing elucidates the molecular mechanism of intragenic copy number variants identified by both array-based and NGS technologies

**Fig. 4:**
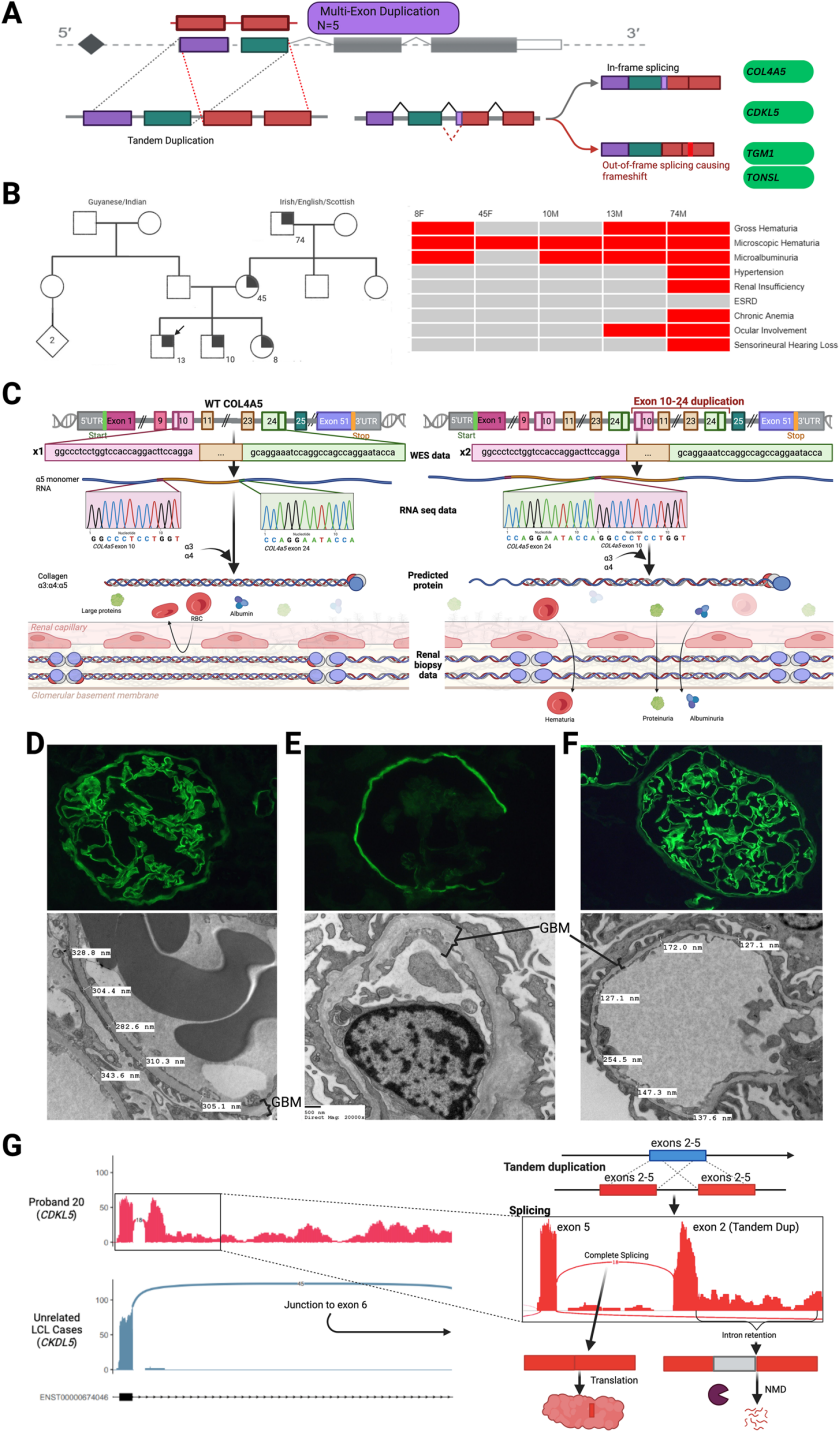
RNA-seq results for probands with candidate copy number variants. **A** Summary of positive RNA-seq results for CNVs. The four variants under investigation (depicted in red) were multi-exon duplications. RNA-seq confirmed all 4 duplications were in-tandem. One led to an in-frame duplication (Case 19), 2 led to out-of-frame aberrant splicing resulting in transcript truncation (Cases 18 and 21), and 1 led to both in-frame and some out-of-frame transcript due to some intron retention (Case 20). **B** Case 19: Proband 19’s pedigree (left) and characteristics of affected family members (right). Arrow indicates the proband. Affected family members are either hemizygous or heterozygous for familial *COL4 A5* variant. **C** Case 19: Proposed hypomorphic mechanism of *COL4 A5* with exon 10-24 duplication. The left panel illustrates how normal Col4a5 joins with collagen alpha-3 and alpha-4 chains to form the glomerular basement membrane (GBM). The right panel shows Proband 14’s intragenic duplication of exons 10-24, confirmed to be in tandem by RNA-seq. The call-out box depicts split-reads found in Proband 19’s RNA-seq using a modified genome backbone harbouring the duplication, which shows in-frame splicing between exons at the border of the tandem duplication. This is predicted to result in an elongated abnormal Col4a5 protein. While this abnormal protein still localizes to the GBM, it causes partial barrier dysfunction is proposed to lead to the proband’s mild phenotype. **D**–**F** Case 19: Proband 19 renal biopsies demonstrate Col4a5 protein stability. Renal biopsies display indirect immunofluorescence staining for type IV collagen α5 chain (superior panel) and electron microscopy (inferior panel) of the glomerulus. **D** Normal control kidney demonstrating normal diffuse linear a5 staining pattern in glomerular basement membranes (GBM), Bowman’s capsule, and distal tubular basement membranes, and intact GBM of normal thickness (~ 307 ± 27 nm) for age-matched controls. **E** Reference male with X-linked Alport syndrome, with global loss of a5 staining in the GBM with preserved staining in Bowman’s capsule and thickening and irregular contours of the GBM with splitting of the lamina densa. **F** Proband 19 with normal diffuse a5 staining and slight thinning of the GBM. **G** Case 20: *CDKL5* Sashimi plots displays tandem duplication of exons in Proband 20 compared to tissue-matched samples, using a modified genome backbone harbouring the duplication (where an additional exon 2-5 sequence was inserted just downstream of the original exon 5 to visualize the split reads spanning the breakpoint). LCL RNA-seq revealed an in-frame, tandem inclusion of the duplicated exons 2-5. However, a significant portion of reads also exhibited intron-retention, which is predicted to be out-of-frame and likely subject to nonsense-mediated decay (NMD)

Next, five unique intragenic copy number variants (CNVs) were investigated through RNA-seq in five probands (please note, Case 18 is also a compound heterozygote with a putative splice variant and is also included in a previous section). We reasoned that RNA-sequencing would help assess if (1) duplications detected by array-based technologies are in tandem and (2) if the impact of these duplications results an in or out-of-frame mRNA transcript (i.e., consistent with that would be expected based on DNA sequence alone). Two variants were classified as VUSs, while three were classified as likely pathogenic, albeit with the three probands having unique phenotypic features providing a rationale for further investigation. The sizes of the duplications ranged from 101 bp to 701 kbp (Table 1 and Additional file 3: Table S2), and all included at least one complete exon (Fig. 4A). RNA-seq was successful for four of the five variants and all exhibited a transcript effect (Cases 18–21). The transcript was confirmed to be in tandem in all 4 cases (by detecting split-reads between exons spanning breakpoints). For two (Case 18, Case 21), the transcripts were out-of-frame. In another case, the transcript was in-frame and resulted in an atypical phenotype (Case 19, further details below). In the fourth case, some in-frame transcript was seen, but there was also some intron retention at the duplication boundary (Case 20). One case was unsuccessful as no reads were seen in the region of the duplication (Case 22). This case was not subjected to further analysis for other candidate variants as maternal tissue was used. All deleterious findings are summarized in Table 1 and detailed in Additional file 4: Table S3. Specific case examples highlighting the utility of RNA-seq for intragenic duplications are highlighted below.

### Case 19—*COL4A5*

Proband 19, a 12-year-old male, presented with thin basement membrane disease with microscopic hematuria, episodic gross hematuria, and mild proteinuria. A targeted array CGH panel revealed a hemizygous multi-exon duplication (spanning exons 10––24) within an X-linked gene, *COL4A5.* The duplication was absent from large population control databases and classified as likely pathogenic. Familial phenotyping revealed that the proband’s sister, brother, mother, and maternal grandfather also exhibited varying degrees of hematuria ± proteinuria (Fig. 4B). All affected family members were tested and found to carry the same multi-exon duplication. Notably, the affected individuals displayed milder renal disease than expected for Alport syndrome which is caused by loss of function variants in *COL4A5*. Interestingly, even the grandfather had not progressed to end stage renal disease (ESRD) as would be expected in this disorder but instead only exhibited mild renal dysfunction (Fig. 4B). RNA-seq on the mother’s skin fibroblasts confirmed that the duplication was in tandem, with splicing occurring between exons 24 and 10, resulting in an in-frame transcript (Fig. 4C). Subsequent research WGS was done to confirm the exact breakpoints of the duplication, which were confirmed to fall within intronic regions (chrX:108,574,886–108,598,389). Antibody staining for the Collagen alpha-5(IV) chain in the proband’s renal tissue was normal, consistent with the production of an abnormal but adequately expressed protein (Fig. 4D–F) causing a likely hypomorphic impact in the proband (Fig. 4C). These findings provide a likely explanation for the mild phenotype in the family and solidified the diagnosis for the proband.

### Case 20—*CDKL5*

Proband 20, male, presented with central hypotonia and infantile spasms. Trio-WGS revealed a de novo intragenic 105 kbp tandem duplication (Xp22,13p22.13(18474185_18579246)), which includes exons 2 (the first coding exon) to 5 of *CDKL5*. CDKL5 deficiency disorder (CDD) is an X-linked developmental and epileptic encephalopathy (DEE) characterized by severe early-onset epilepsy and motor, cognitive, visual, and autonomic disturbances [55]. This proband presented with an atypical phenotype for a male, with typical development until infantile spasm onset 9 months of age [56]. While the DNA variant was classified as likely pathogenic, the atypical phenotype furthered diagnostic uncertainty. RNA-seq performed on LCLs confirmed that the duplication was in-tandem, with splicing occurring between exons 5 and exon 2, resulting in an in-frame transcript. Low levels of intron retention was seen at the duplication boundary, which likely triggers some level of NMD (Fig. 4G). These results suggest that the tandem duplication disrupts only a minority of transcripts with the rest encoding an in-frame likely functional protein, providing an explanation for the proband’s milder phenotype.

### Variants in the untranslated regions can impact the RNA transcript through multiple molecular mechanisms

**Fig. 5:**
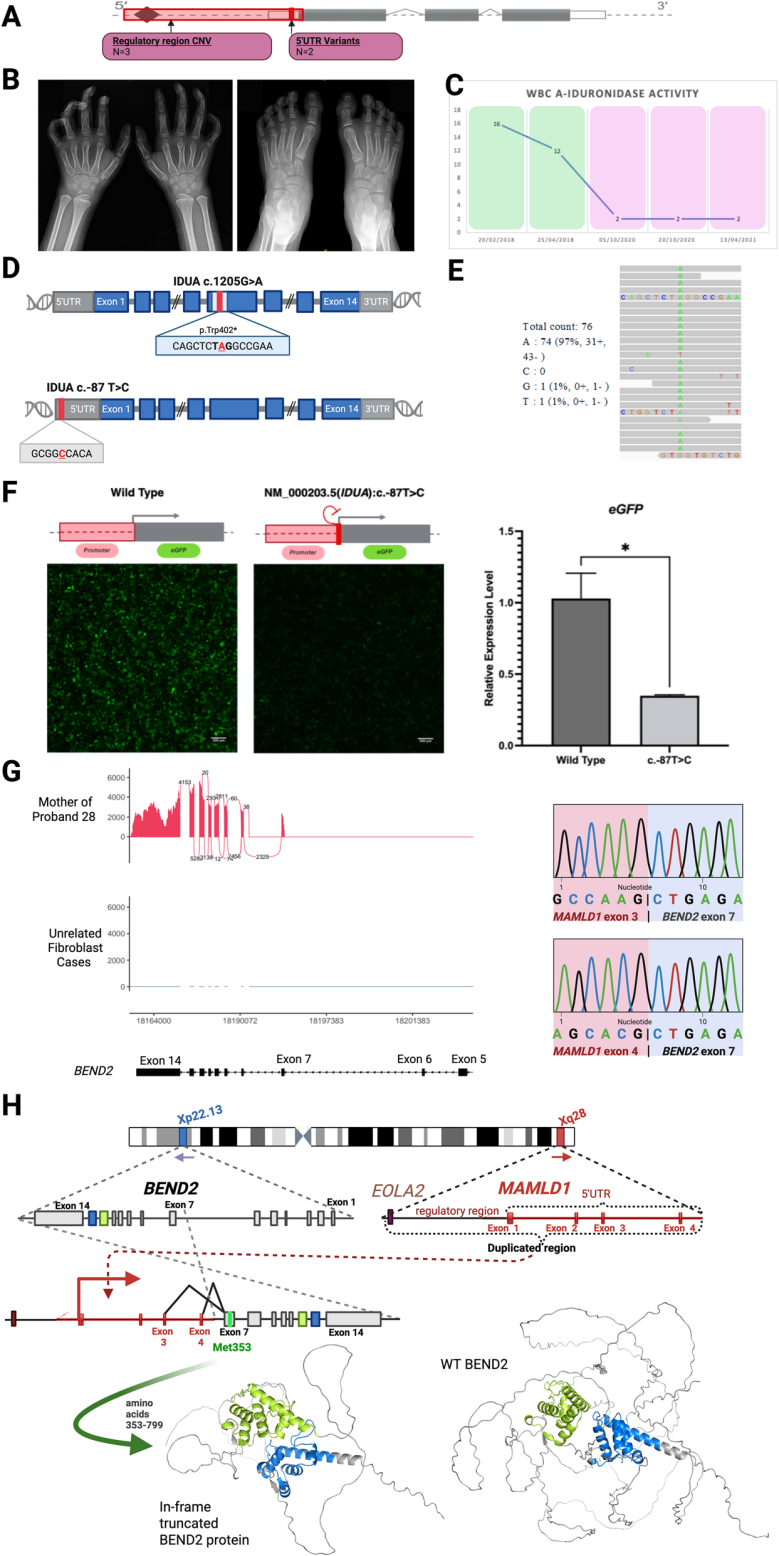
RNA-seq results for probands with candidate regulatory non-coding variants. **A** Summary of positive RNA-seq results for regulatory variants (red). Two were missense variants in the 5′UTR, while three were copy number variants (two duplications and one deletion) affecting the 5′UTR and upstream regulatory region. RNA-seq demonstrated that one of the 5′UTR variants led to the repression of transcription (Case 24), while one of the duplications resulted in upregulation of another distant gene (Case 28). **B** Case 14: Proband 14’s bilateral hand and foot X-rays demonstrating diffuse osteopenia, sclerotic lucencies along the midshaft regions of the proximal phalanges with squared or under-tubulation remodelling and coarse appearance of the medullary zone of the small bones of the hand. There are acro-osteolysis again noted involving the tufts of the distal phalanges. **C** Case 14: Results of in vitro functional testing for MPS-1; α-L-iduronidase enzyme activity assays. Activity is expressed as nanomoles of phenol liberated during 18-h incubation per mg of protein. Times when the proband was receiving ERT are highlighted in green, and times without ERT are in red. **D** Case 14: Depiction of Proband 14’s compound heterozygous *IDUA* variants. The superior panel demonstrates the pathogenic c.1205G > A variant which results in introduction of an early stop codon and is predicted to result in nonsense-mediated decay. The inferior panel demonstrates the c.-87 T > C in the 5’UTR, located at the + 2-position relative to the transcription start site, suggesting its role in in downregulating transcription. **E** Case 14: *IDUA* RNA-seq in LCLs from Proband 14 demonstrates 97% skew towards the allele harboring the known pathogenic variant, resulting in the predominance of the wild-type A nucleotide (corresponding to amino acid 402). **F** Case 14: HEK-293 cells transfected with the patient variant demonstrated decreased promoter efficiency compared with wild type IDUA promoter region. The left panel shows EGFP signal from cells transfected with wild type IDUA promoter in contrast to the right panel showing EGFP signal from cells transfected with probands 14’s IDUA promotor affected by the c.-87 T > C 5′UTR variant. eGFP gene expression level from RT-qPCR are compared in the center bar graph (mean ± SEM; *: *P* < 0.05). **G** Case 28: Sashimi plots display expression of *BEND2* in fibroblasts from the mother of Proband 28, highlighting the *MAMLD1* regulatory region, compared to controls. RNA-seq from fibroblasts reveals multiple split reads, with the two most prominent mapping from a duplicated non-coding exons 3 and 4 of the *MAMLD1* 5′UTR to coding exon 7 of the distant *BEND2* gene, located on the other end of the X-chromosome. The details of the split read sequences are depicted in the right panel. **H** Case 28: This panel illustrates a proposed molecular mechanism of the *MAMLD1-BEND2* fused transcript, inferred from the RNA-seq analysis of Proband 28. It is hypothesized that the *MAMLD1* duplication has been inserted upstream of *BEND2*, likely upstream of its exon 7. This insertion may lead to the activation of a truncated BEND2 protein expressed under regulation of the *MAMLD1* promoter and 5’UTR. The truncated protein is predicted to be translated from a downstream start codon (Met 353), causing N-terminal truncation but thereby keeping the two downstream functional BEND domains intact. This is demonstrated by the below Colabfold protein model [34] predicting folding of proband 28’s truncated BEND2 (446 amino acids), where the 2 BEND functional domains are depicted in blue and green. The model to the right shows the reference wildtype BEND2 protein model with an intact N-terminal end (799 amino acids)

Five variants affecting the 5′ untranslated regions of genes felt to match their phenotypes were identified in five probands. All five were classified as VUSs. Two variants were single base substitutions (Cases 24 and 26) (Fig. 5A). Three variants were CNVs; a deletion that encompassed the region upstream of the gene, the first (non-coding) exon which included the 5′UTR, part of the first intron (Case 25), and two duplications involving the 5′UTR and promoter regions (Case 28, Case 27) (Fig. 5A). RNA-seq was successful in confirming or refuting a transcript effect for all five variants. Two cases were positive for a transcript effect (see details below); in one, allele bias was seen, which confirmed the diagnosis (Case 24), and in another a possible gene fusion event increased diagnostic suspicion, although further investigation is required (Case 28) (Fig. 5A). The other three cases (Cases 25, 26, and 27) were negative, where no significant effect on expression or splicing was observed, allowing the diagnosis to be ruled out. For one of these probands, duplication of the 5′UTR appeared in tandem but did not affect transcript splicing or expression levels (Case 27). For the three negative cases, proband RNA-seq data was further analyzed to assess for any alternate putative disease-causing aberrations. All deleterious findings are summarized in Table 1 and detailed in Additional file 4: Table S3. Further details regarding highlighted cases are below.

### Case 24—*IDUA*

Proband 24, a 3-year-old male, exhibited musculoskeletal features consistent with a mild MPS-1 phenotype (i.e., Scheie disease). His symptoms included early carpal tunnel syndrome, contractures, arthropathy, myotendinitis, course appearing small bone medulla, and osteopenia (Fig. 5B). Initial *IDUA* (NM_000203.5) sequencing revealed only a heterozygous pathogenic nonsense variant, c.1205G > A (p.Trp402 Ter). This alone was insufficient to confirm a diagnosis for a recessive condition. Enzymatic functional testing was low normal while on enzyme replacement therapy (ERT), and when stopped due to insurance issues, returned to low levels consistent with mild MPS-1 disease (Fig. 5C). Subsequent trio-ES identified a novel heterozygous VUS (c.−87 T > C) in the 5′UTR of *IDUA*, inherited in trans with the pathogenic missense variant (Fig. 5D). Notably, the 5′UTR variant alters the second base pair within the highly conserved transcription start site (TSS) region [57, 58] (Fig. 5D). RNA-seq revealed significant allelic imbalance, with the pathogenic allele accounting for 97% of transcripts (Fig. 5E). This suggests significantly reduced expression from the allele expressing the 5′UTR variant. To directly investigate the molecular mechanism, HEK293 were transfected cells with a plasmid containing the *IDUA* promoter, including the TSS sequence, upstream of an *eGFP* reporter, with and without the patient’s 5′UTR variant (Fig. 5F). While control cells showed robust *eGFP* expression, cells transfected with the plasmid carrying the patient’s variant showed significantly decreased mRNA expression and EGFP fluorescence (Fig. 5F), consistent with transcriptional disruption caused by this variant. Altogether, these results were considered diagnostic for the proband.

### Case 28—*MAMLD1*

Proband 28 presented with left-sided congenital diaphragmatic hernia, hypospadias, and suspected micropenis. Microarray revealed a novel maternally inherited 407 kb X chromosome duplication, classified as a VUS. This duplication encompasses the 5’UTR and regulatory region of *MAMLD1*, extending into the upstream *EOLA2* gene. Loss-of-function variants in *MAMLD1* cause X-linked hypospadias in males (no associated phenotypes in females), raising the possibility that this variant may be disease-causing (Online Mendelian Inheritance in Man ID: 300,758). RNA-seq analysis on fibroblasts from the proband’s mother, who is homozygous for the variant, revealed split reads mapping from the duplication breakpoint downstream of the MAMLD1 regulatory region to a middle exon of the *BEND2* gene (Fig. 5G). *BEND2*, a known chromatin regulator [59], has been identified as an oncogenic driver in recurrent pediatric neuro-epithelial tumors. When fused with regulatory regions of other genes, such as the *MAMLD1* promoter [59], these regions have been shown to promote the expression of *BEND2* [60]. Typically, *BEND2* is not expressed in fibroblasts and is primarily expressed in the testes, spleen, and bone marrow [19]. In contrast, *MAMLD1* has a much more widespread expression pattern [19]. Nevertheless, RNA-seq analysis revealed robust expression of the second half of the *BEND2* transcript in fibroblasts, where split reads demonstrated that it is fused to the non-coding 5′UTR exons of *MAMLD1*. *BEND2* expression was not seen in any other internal fibroblast cases. This suggests that an in-frame *BEND2* protein with a truncated N-terminus is being specifically expressed in the context of a *MAMLD1* regulatory region and 5′UTR (expressed in the developing genitalia) (Fig. 5H). This is hypothesized to cause a gain-of function effect of *BEND2* and resulting in hypospadias in the proband; however, additional functional studies are required for confirmation. Therefore, the clinical diagnostic implications of this variant’s RNA-seq results remains uncertain.

### Low utility of RNA-seq in patients with uninformative whole genome sequencing

Twenty individuals were recruited for RNA-seq who had undergone trio (or in one case, quad) WGS that was negative for any reportable DNA-variants of interest. One proband had a known molecular diagnosis of a chromosome 3p26.1 deletion syndrome, which only partially explained their phenotype, while all other patients had no molecular diagnoses for any of their features. Tissue choice was selected based on patient phenotypes and sample accessibility (Fig. 1B). This included five fibroblast samples (26%), six whole blood samples (32%), five cultured LCLs samples (26%), and four muscle samples (21%, Fig. 1B, Additional file 3: Table S2). In one case (Case 35), singleton RNA-seq was conducted on muscle biopsies from an affected sibling as a biopsy was declined for the proband. In addition to these 20 cases, we also subsequently included 15 probands from our cohort above with candidate DNA variants who did not receive a diagnosis after RNA-seq was used to evaluate the impact of the variant in question. All 35 cases were evaluated for abnormal splicing and expression outliers as described in the Methods. From this analysis a single putative RNA candidate (Case 44) was identified (1/35, 3%). Proband 44 is a male who presented with postnatal growth delay, symmetric short stature, hypoplastic optic nerve, distinctive facial features, global developmental delay, and autism spectrum disorder. RNA-seq analysis identified a novel, out-of-frame skipping of exon 67 in 26% of reads, that was absent from internal controls and GTEx (Additional file 1: Fig. S1 C). Hemizygous loss-of-function variants in *HUWE1* are associated with a X-linked syndromic intellectual developmental disorder, Turner type (OMIM# 309,590), associated with features overlapping the probands including short-stature/growth delay, autistic features, distinctive features and other neurologic concerns. This finding prompted a re-analysis of the *HUWE1* gene in the clinical genome which revealed a maternally inherited variant in intron 67, c.10035 + 21 T > A, in *HUWE1* (NM_031407.7) that was initially not reported due to a lack of predicted impact on splicing from in silico tools. While there is evidence that hypomorphic variants in *HUWE1* cause disease [61], whether the level of transcript disruption observed is sufficient to be pathogenic remains uncertain.

## Discussion

**Fig. 6:**
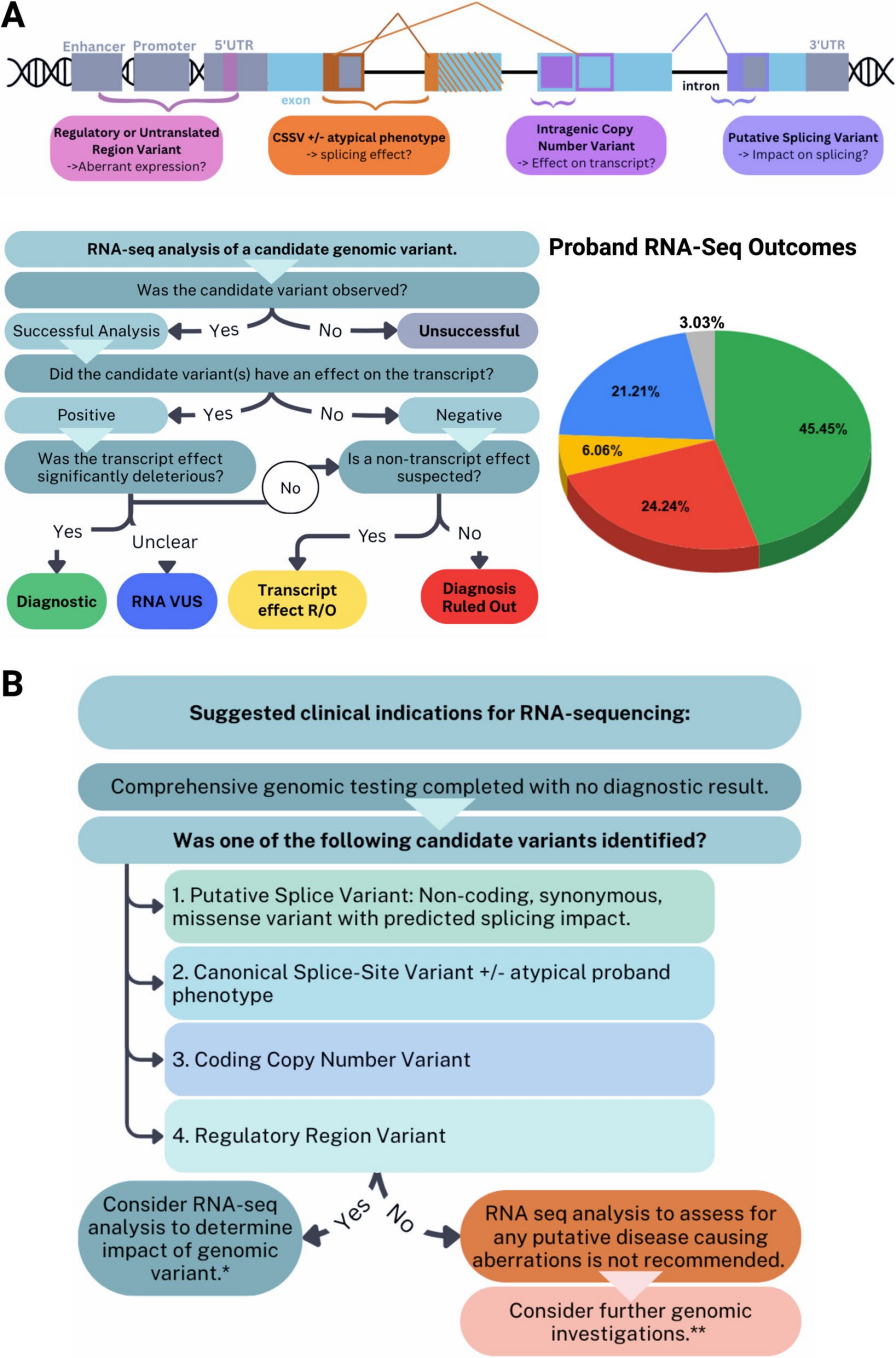
Summary of study outcomes. **A** Summary of the candidate variants examined by RNA-seq and their observed impact. Clinical diagnostic outcomes for the candidate variants are demonstrated in the pie chart, where colour corresponds to the outcome as indicated in the legend; green indicates a diagnostic outcome for the proband, red indicates the diagnosis was ruled out, the other results indicate ongoing diagnostic uncertainty due to either an RNA VUS (blue), a result ruling out a transcript effect in context of another possible mechanism of pathogenicity (yellow) or an unsuccessful result (grey). See also Figure S1 for Sashimi plots for all probands with positive RNA-seq results not highlighted in the main text, and Additional file 3: Table S2 for detailed summaries of all probands’ RNA-seq findings. **B** Suggested approach for the clinical application of RNA-seq in patients with suspected Mendelian disease. *RNA-seq should be conducted on tissue where adequate expression of the target gene of interest has been confirmed. **Alternate genomic investigations may include broader genome sequencing, WES/WGS re-analysis, refined tests such as methylation or repeat expansion studies, and long-read genomic sequencing. Furthermore, functional assays or other research testing may be considered

In this study, we find that RNA-seq achieves a high diagnostic yield when used to specifically evaluate candidate DNA variants predicted to impact the RNA transcript in four specific and common clinical scenarios. RNA-seq analysis provided a molecular diagnosis in 45% of the 33 individuals with candidate variants and helped exclude a candidate variant in an additional 24% for a combined diagnostic utility of 69% (Fig. 6A). These findings align with previous work demonstrating that RNA-seq analysis can reclassify a large proportion (75%) of putative splicing variants [52], emphasizing its utility in clarifying WGS data. Furthermore, there was an improvement in candidate variant resolution (ie. increased suspicion of pathogenicity of a given variant) in an additional 21% of probands, which included two new putative disease genes (*MED14* and *PPP1R2)*. In contrast, in the 35 patients in which RNA-seq was performed without a candidate DNA variant (or to look for alternate candidates following non-diagnostic hypothesis-driven RNA-seq), only a single potential diagnosis was identified (3%). Overall including both hypothesis-driven and hypothesis independent analysis, diagnostic utility was 43% (Diagnostic and Diagnosis Ruled Out outcomes). In addition to a high diagnostic utility when applied to candidate DNA variants, our study identified multiple novel mechanisms of disease. Understanding these novel mechanisms are essential not just to understand unique clinical phenotypes but are also essential as we move into the era of precision genetic medicines.

This study has refined the molecular scenarios in which clinicians should pursue RNA-seq as a diagnostic test (Fig. 6B). The findings suggest that RNA-seq is most effective as an adjuvant diagnostic test, when a candidate variant has already been identified that may affect the mRNA transcript. While there are likely other clinical scenarios in which RNA-seq analysis may be helpful, we suggest four categories of variants that RNA-seq can further clarify pathogenicity: (i) a putative splice variant outside of the canonical splice sites, (ii) a canonical splice site variant where a patient is presenting with an atypical phenotype or a new disease gene is being established, (iii) an intragenic copy number variant, or (iv) a variant within regulatory elements or genic untranslated regions. If a patient lacks a candidate variant, we suggest pursuing additional DNA-based testing before considering RNA-seq. These findings are consistent with a previous study by our group, which examined an additional cohort of 40 probands with suspected monogenic disease [28]. While several disease-causing variants were identified through an “RNA-first” approach (blinded to genomic variants already identified), all variants had already been detected through GS. Although there are theoretical scenarios in which RNA-seq may reveal a pathogenic aberration in the absence of an obvious DNA candidate variant on prior testing, such as when a clear clinical diagnosis with limited locus heterogeneity has been made, these instances are nonetheless likely limited by the low signal-to-noise ratio of current testing, making it challenging to identify any candidate RNA-level aberrations. This low signal-to-noise ratio likely explains why we only observed a diagnostic yield of 3% in patients without a previously identified candidate variant. We should also note that this low diagnostic yield may also be influenced by our specific analysis pipeline, the tissue selected, the patient cohort and the previous DNA testing that was completed. Altogether, our findings suggest that RNA-seq may be most effective as a second-tier test, although this may depend on factors mentioned earlier and evolve with advancements in technology and analysis in the future.

In this study, RNA-seq was found not only to be effective for evaluating the pathogenic impact on RNA transcripts, but also for elucidating the molecular mechanism of disease. Understanding these mechanisms directly impacts clinical care. When a variant is associated with an atypical phenotype, clinicians may question whether it fully accounts for the patient's clinical presentation. This uncertainty is particularly pronounced for CSSVs, where significant discrepancies have been observed between in silico predictions of pathogenicity and functional evidence [48]. Such ambiguity can result in unnecessary additional diagnostic testing and increased patient anxiety. Confirming and clarifying the molecular mechanism adds confidence in the clinical diagnosis. It is also essential for the development of targeted therapies, such as antisense oligonucleotide (ASO) design. Novel molecular mechanisms of disease were uncovered in 30% of probands, involving either established or candidate genes. Examples of these mechanisms include the following: (1) the generation of an in-frame transcript causing a truncated protein with likely novel function (*TBX6*), (2) an in-frame transcript causing a likely hypomorphic protein (*CDKL5, COL4 A5, SASS6*), (3) a leaky splice site causing an out-of-frame transcript that only impacts a minority of transcripts, resulting in a reduction, but not complete haploinsufficiency (*EFTUD2, MED14, PP1PR2*), (4) a variant adjacent to the transcriptional start site causing mono allelic expression and skew towards the pathogenic allele (*IDUA*), and (5) an isoform switch resulting in the expression of proteins that are normally only expressed at very low levels (*NFU1*). Novel mechanisms were uncovered across all four categories of cases assessed, demonstrating the broad utility of RNA-seq. Importantly, novel mechanisms of disease were even uncovered in patients with canonical splice site variants (*EFTUD2, TBX6, SYNGAP1*), reinforcing the value of RNA-seq even when a variant has sufficient evidence to be classified as pathogenic or likely pathogenic.

While our overall cohort size of 53 unrelated individuals is comparable to other rare disease RNA-seq studies, we acknowledge that the small sample sizes within variant-specific subgroups, particularly for extremely rare classes like intragenic duplications and 5′UTR variants, limit the generalizability of diagnostic rates. Further study of these subgroups will be required to better understand the utility of RNA-seq in these scenarios. Intragenic CNVs specifically have received minimal attention in routine clinical RNA-seq applications, leaving gaps in our understanding of their functional impact. While some tools have been developed to detect complex rearrangements including duplications [53], standardized clinical approaches for intragenic CNV characterization remain limited. For duplications not identified on a whole genome sequencing backbone (i.e., microarray or exome), determining whether a duplication is in-tandem or located elsewhere in the genome is crucial to interpret the potential impact on transcripts. RNA-seq can confirm that a duplication is in tandem by identifying novel split reads that bridge two exons (at the most 5′ and most 3′ exons at either end of the duplication) that are normally not adjacent (as seen in cases *TGM1, COL4 A5, CDKL5, TONSL*). Another useful application, even in the context of existing WGS, is verifying whether a tandem duplication results in proper splicing. A tandem duplication creates novel a genomic context with altered intronic and exonic splice enhancers and inhibitors. Therefore, it cannot be assumed that splicing will align one exon in-frame with the next exon, especially at the borders of the duplication. This was demonstrated in one case, where the tandem duplication not only created an in-frame transcript, but also resulted in intron retention (*CDKL5*). This suggests that the presence of this tandem duplication led to some clinically significant abnormal splicing, which was not predicted based on sequence alone.

One case (*MAMLD1*) identified in this study highlights a unique application of RNA-seq, revealing an entirely unexpected putative molecular mechanism. Whole genome sequencing identified a duplication spanning the promoter of *MAMLD1*. RNA-seq revealed (Fig. 5G) that this duplicated sequence inserted on the opposite end of the X-chromosome within the *BEND2* gene, resulting in the activation of a truncated BEND2 protein. While *MAMLD1* is broadly expressed across tissues, *BEND2* is not normally expressed in fibroblasts [19] suggesting that its expression in the proband’s mother’s fibroblasts is entirely driven by this novel event. Interestingly, although the *MAMLD1* promoter was inserted within *BEND2*, the resulting fusion transcript is expected to lead to transcription of a truncated BEND2 protein that still includes both “BEND” functional domains (Fig. 5H). While whether this truncated protein is functional is yet to be determined, multiple reports indicate that BEND2 proteins with truncated N-terminuses have been found in various types of tumours, suggesting that this protein is likely functional. BEND2 is known to play a role in chromatin regulation but remains poorly studied. We hypothesize that the expression of this truncated protein driven by the *MAMLD1* regulatory region (which is expressed in the developing genitalia) results in a gain-of-function causing hypospadias in this patient. This case provides evidence of a potential germline fusion causing Mendelian disease, illustrating a novel molecular mechanism typically only seen somatically in cancer. This case also highlights the importance of RNA-seq in clarifying the impact of non-tandem duplications that may integrate into various regions of the genome. The successful analysis of 97% of candidate variants, despite a predominance of neurologic phenotypes demonstrates that prioritization of candidate genes to identify appropriate tissue sources can enhance the efficacy and diagnostic yield of RNA-seq.

Despite the successes of RNA-seq analysis for the resolution of candidate variants in this rare disease cohort, several limitations remain. While expression outliers were assessed, the pipeline relied on GTEx as a reference, which may not be optimal for pediatric samples. The current analysis method did not account for variables such as age or technical variability in sequencing and experimental protocols. Moving forward, constructing an internal reference cohort will be an important step to enable a more comprehensive analysis of expression outliers. Additionally, this study illustrates that detecting significant RNA-level changes does not always clarify pathogenicity, resulting in RNA-level VUSs (21%). For example, in Case 11, a non-coding variant led to an in-frame exon extension in 22% of transcripts. The reduced percentage of transcripts impacted, combined with the in-frame consequence, made it very challenging to conclude whether this variant was disease-causing. Similarly, in Case 10, involving compound heterozygous splicing variants located close to one another, it was difficult to determine which variant caused the specific splicing alterations observed. Furthermore, application of RNA-seq data to current ACMG functional studies criteria (PS3, PM1, BS3) for variant classification remains subjective. [62] We emphasize that even after analysis of both DNA and RNA, complementary functional tests are sometimes required to confirm pathogenicity. Emerging long-read RNA-seq techniques may offer a resolution for some of these challenges [63].

## Conclusions

RNA-seq showed strong utility in an undiagnosed rare-disease cohort as an ancillary tool for evaluating candidate DNA variants suspected to affect RNA, enabling confirmation or exclusion of a molecular diagnosis in 69% of cases. It also revealed novel disease mechanisms, key to understanding complex phenotypes and informing targeted management. These findings support integrating RNA-seq into clinical workflows to improve diagnostic yield beyond whole genome sequencing and highlight the need for its standardization and broader clinical adoption in rare disease care.

## Supplementary Information

Additional file 1. Fig. S1: Sashimi plots for all cases with positive RNA-seq findings not highlighted in the main text (related to Table 1 and Fig. 6).

Additional file 2. Table S1: Summary of relevant journal articles where RNA-seq was used in various rare disease populations.

Additional file 3. Table S2: Summary of cohort composition and demographics (related to Fig. 1A).

Additional file 4. Table S3: Summary of All Probands Phenotypes and RNA-seq Results (related to Table 1 and Fig. 6).

## Data Availability

Plasmids produced in this study are available upon request. Relevant genotype-phenotype data are provided in the text, while RNA sequencing data cannot be shared because of patient confidentiality, as the study protocol permits the publication of findings and results but does not allow for the sharing of raw data. Additional data not included in the manuscript may be requested from the corresponding author. All code used in the analysis is released at: 10.5281/zenodo.15212187 [29]. Requests for further information and resources should be directed to and will be fulfilled by the corresponding author and lead contact, Dr. Ashish Deshwar (ashish.deshwar@sickkids.ca).
