# Supplementary material for: Clinical applications of and molecular insights from RNA sequencing in a rare disease cohort
Source: Genome Med. 2025 Jul 1;17:72. doi: 10.1186/s13073-025-01494-w (PMC12210447; doi:10.1186/s13073-025-01494-w)
Supplement: Supplementary file 1 — Additional file 1. Fig. S1: Sashimi plots for all cases with positive RNA-seq findings not highlighted in the main text (related to Table 1 and Fig. 6). [file 13073_2025_1494_MOESM1_ESM.docx]

**Fig. S1: Sashimi plots for cases with positive RNA-seq findings not highlighted in the main text.**

S1A) Case 3: Sashimi plot of *PPP1R2* RNA-seq in blood demonstrating skipping of exon 4 in some reads (red) resulting from the c.403+3A>T *PPP1R2* variant in Proband 3, in contrast to normal splicing seen in unrelated tissue-matched samples (blue).

S1B) Case 15: *MED14* sashimi plots in Proband 15 compared to tissue-matched unrelated samples. Proband 15’s LCL RNA-seq results at the *MED14* locus (red) displays an out-of-frame loss of the C-terminal end of exon 18 due to novel splice donor use in 1.72 percent of reads as a result of the c.2365+2T>C variant, compared to unrelated LCL RNA-seq cases where all 990 reads display normal splicing (blue).

S1C) Case 44: *HUWE1* Sashimi plots display out-of-frame exon skipping in Proband 44, with no candidate variant previously identified on whole genome sequencing. The superior panel displays the proband’s blood RNA-seq results at the *HUWE1* locus (red) which showcases novel out-of-frame skipping of exon 67 in 26% of reads, compared to unrelated blood RNA-seq cases where all reads display normal splicing (blue).

S1D) Case 8: *PIEZO1* Sashimi plots display out-of-frame exon 21 extension by 5 nucleotides in all reads in Proband 8’s blood at the *PIEZO1* locus (red) due to the c.2991+7C>T, compared to unrelated blood RNA-seq cases where all reads display normal splicing (blue).

S1E) Case 9: *ELN* Sashimi plots display multiple abnormal splice junctions near the c.1719 T>A variant site, likely resulting in NMD, in Proband 9’s fibroblasts at the *ELN* locus (red). This likely results in NMD, as supported by over 90% skew to the reference allele. This is compared to unrelated fibroblast RNA-seq cases where all reads display normal splicing (blue).

S1F) Case 10: *FOXRED1* Sashimi plots display exons 5 and 6 and resultant intron retention in Proband 10’s blood at the *FOXRED1* locus (red) and showcases that both the c.536+5G>A candidate splice donor variant, and the c.733+1G>A pathogenic splice donor variant contribute to this. This is compared to unrelated blood RNA-seq cases where all reads display normal splicing (blue).

S1G) Case 11: *PQBP1* Sashimi plots display in-frame exon 4 extension by 12 nucleotides in 22% of transcripts extension and additional minor intron retention in Proband 11’s blood at the *PQBP1* locus (red) as a result of the c.292+5G>A variants’ creation of a novel splice donor. This is compared to unrelated blood RNA-seq cases where all reads display normal splicing (blue).

S1H) Case 12: *PIEZO2* Sashimi plots displays how the c.7743-8A>G variant’s introduction of a novel splice acceptor causes out-of-frame exon 54 extension by 7 nucleotides, seen in all reads, and intron retention in Proband 12’s LCLs at the *PIEZO2* locus (red). There is also intron 53 retention and loss of exon 53 and 54 expression in 50% of reads. Unrelated LCL RNA-seq cases are compared, where all reads display normal splicing (blue).

S1I) Case 17: *SYNGAP* Sashimi plots display out-of-frame 13bp shortening of exon 12 at the *SYNGAP* locus (red) in Proband 17’s LCLs, as a result of the c.1914-1G>C variant. Skew is 77% toward the reference allele. This is compared to unrelated LCL RNA-seq cases where all reads display normal splicing (blue).

S1J) Case 18: *TGM1* Sashimi plots display the effects of two variants at the TGM1 locus (red) in Proband 18’s skin. The maternally inherited duplication of exons 10–14 is in-tandem and out-of-frame, with intron retention observed beyond the duplication boundary. There is out-of-frame skipping of exon 7 in 50% of transcripts resulting from the *de novo* splice acceptor variant c.985-3C>G in intron 6. There is no internal control for skin.

S1K) Case 21: *TONSL* Sashimi plots display the maternally inherited duplication of exon 25 is in-tandem and out-of-frame, resulting in a frameshift at the TONSL locus (blue) in Proband 21’s mother’s LCLs. No duplication is seen in Proband 21’s LCLs (red), possibly due to loss of the variant during cell culture.

S1L) Case 51: *FBXL4* Sashimi plots display the c.1703-4A>G VUS results in intron 9 retention in ~75–80% of transcripts at the FBXL4 locus in Proband 51’s fibroblasts (red), compared to much lower levels in fibroblast internal control samples (blue).

S1M) Case 52: *ACADM* Sashimi plots display the synonymous c.85C>A; p.Arg29= variant results in skipping of canonical exon 2 in ~50% of reads at the ACADM locus in Proband 52’s whole blood (red), leading to frameshift. This is compared to minimal exon 2 skipping in whole blood RNA-seq control samples (blue).

S1N) Case 53: *UFC1* Sashimi plots display the c.333-14T>C VUS causes a 10-fold increase in exon 5 skipping (300 reads, vs 30 reads in whole blood internal controls (blue)) at the UFC1 locus in Proband 53’s whole blood (red), reducing exon 5 expression by 25%.
