# Supplementary material for: Clinical applications of and molecular insights from RNA sequencing in a rare disease cohort
Source: Genome Med. 2025 Jul 1;17:72. doi: 10.1186/s13073-025-01494-w (PMC12210447; doi:10.1186/s13073-025-01494-w)
Supplement: Supplementary file 2 — Additional file 2. Table S1: Summary of relevant journal articles where RNA-seq was used in various rare disease populations. [file 13073_2025_1494_MOESM2_ESM.docx]

**Table S1: Summary of relevant journal articles where RNA-seq was used in various rare disease populations**. N indicates the number of undiagnosed probands for which RNA-seq was done. The majority have only used broad transcriptome-wide analysis (transcriptome-first hypothesis-independent approach).

| **Article** | **Target Population** | **N Undiagnosed Patients** | **Tissues Sampled** | **Analysis Approach** | **RNA-seq Finding Rate** |
| --- | --- | --- | --- | --- | --- |
| **Cummings et al., Sci. Transl. Med. 2017**(6) | Rare muscle disorders | 50 | Muscle | Transcriptome-first and genome-first | 35% |
| **Kremer et al., Nat. Commun. 2017**(2) | Mitochondrial disease | 48 | Fibroblasts | Transcriptome-first | 10% |
| **Fre ́sard et al., Nat. Med. 2019**(58) | Mendelian disease | 80 | Blood | Transcriptome-first | 7.50% |
| **Gonorazky et al., Am. J. Hum. Genet. 2019**(1) | Neuromuscular disorders | 25 | Blood, transdifferented myotubules | Transcriptome-first | 36% |
| **Wai et al., Genet. Med. 2020**(59) | Mendelian disease | 17 | Blood | Transcriptome-first | 24% |
| **Lee et al., Genet. Med. 2020**(60) | Mendelian disease | 48 | Blood, fibroblasts, muscle, bone marrow | Transcriptome-first | 18% |
| **Murdock et al., J. Clin. Invest. 2021**(61) | Mendelian disease | 82 | Blood, fibroblasts | Transcriptome-first | 17% |
| **Ye ́pez et al., Genome Med. 2022**(3) | Mendelian disease, primarily mitochondrial disease | 205 | Fibroblasts | Transcriptome-first | 16% |
| **Bournazos et al., Genet. Med. 2022**(62) | Mendelian disease | 19 | Blood, EBV-LCLs, fibroblasts, urothelial cells | Genome-first | 40% |
| **Dekker et al., Am. J. Hum. Genet. 2023**(63) | Neurodevelopmental disorders | 67 | Fibroblasts | Transcriptome-first | 13% |
| **Deshwar et al., Am. J. Hum. Genet. 2023**(43) | Mendelian disease | 39 | Blood | Transcriptome-first | 8% |
| **Oquendo et al., Genome Med. 2024**(5) | Mendelian disease | 80 | Blood | Transcriptome-first | 23% |
