# Supplementary material for: Clinical applications of and molecular insights from RNA sequencing in a rare disease cohort
Source: Genome Med. 2025 Jul 1;17:72. doi: 10.1186/s13073-025-01494-w (PMC12210447; doi:10.1186/s13073-025-01494-w)
Supplement: Supplementary file 3 — Additional file 3. Table S2: Summary of cohort composition and demographics (related to Fig. 1A). [file 13073_2025_1494_MOESM3_ESM.docx]

**Table S2: Cohort Composition and Demographics.** First table subheading shows clinical features observed among the 39 probands with multisystem involvement. These individuals may be represented in multiple categories based on their phenotypic complexity. Second subheading demonstrates clinical features of the 14 probands with single system involvement where each individual is categorized by the predominant system affected. Final three subheadings summarize how many probands had candidate variants versus those without, the types of prior genetic testing used in cases with candidate variants (aCGH indicates array comparative genome hybridization), and the tissue types used for RNA-seq. Percentages are relative to the respective subgroup totals.

| **Clinical Feature** | **N** | **Percentage** |
| --- | --- | --- |
| **Multisystem** | **39** | **74%** |
| Neurologic | 24 | 61.50% |
| Global Developmental Delay / Intellectual Disability (± Autism) | 20 | 51.30% |
| Dysmorphic features | 18 | 46.20% |
| Multiple Congenital Anomalies | 13 | 33.30% |
| Ocular | 12 | 30.80% |
| Growth abnormalities | 11 | 28.20% |
| Endocrine | 11 | 28.20% |
| Cardiac | 7 | 17.90% |
| Respiratory | 6 | 15.40% |
| Gastrointestinal | 6 | 15.40% |
| Hematologic/Immunologic | 6 | 15.40% |
| Skeletal | 5 | 12.80% |
| Hearing Loss | 4 | 10.30% |
| Musculoskeletal | 4 | 10.30% |
| Neuromuscular | 3 | 7.70% |
| Dermatologic | 3 | 7.70% |
| Renal | 1 | 2.60% |
| Urogenital | 1 | 2.60% |
| Rheumatologic | 1 | 2.60% |
| **Single System** | **14** | **26%** |
| Neuromuscular | 3 | 21% |
| Neurologic | 3 | 21% |
| Skeletal | 2 | 14% |
| Metabolic | 1 | 7% |
| Global Developmental Delay / Intellectual Disability (± Autism) | 1 | 7% |
| Musculoskeletal | 1 | 7% |
| Renal | 1 | 7% |
| Dermatologic | 1 | 7% |
| Non-immune Hydrops | 1 | 7% |
| **Proband Cohort Group** | | |
| With Candidate Variant | 33 | 62% |
| Without Candidate Variant | 20 | 38% |
| **Prior Genetic Testing of Cohort with Candidate Variant** | | |
| WGS Only | 13 | 39% |
| WES Only | 10 | 30% |
| Both WES and WGS | 6 | 18% |
| Trios Among Above | 25 | 86% |
| Microarray / Panel / aCGH | 4 | 12% |
| **RNA-seq Tissue Type** | | |
| Lymphoblastoid Cell Lines (LCLs) | 11 | 33% |
| Fibroblasts | 11 | 33% |
| Whole Blood | 10 | 30% |
| Skin (Direct Biopsy) | 1 | 3% |
| Parent/Sibling Fibroblast | 3 | 9% |
