# Supplementary material for: Clinical applications of and molecular insights from RNA sequencing in a rare disease cohort
Source: Genome Med. 2025 Jul 1;17:72. doi: 10.1186/s13073-025-01494-w (PMC12210447; doi:10.1186/s13073-025-01494-w)
Supplement: Supplementary file 4 — Additional file 4. Table S3: Summary of All Probands Phenotypes and RNA-seq Results (related to Table 1 and Fig. 6). [file 13073_2025_1494_MOESM4_ESM.docx]

**Table S3: Summary of All Probands Phenotypes and RNA-seq Results.** The first column lists the gene, Phenotype Online Mendelian Inheritance of Man (OMIM) number for known disease genes, and transcript of interest. The second column details the genomic variant of interest (in bold), as well as *in trans* variants (not under investigation) for recessive conditions (non-bold). It also includes the variant classification and variant inheritance (maternal (mat), paternal (pat) or *de novo*). The third column denotes key phenotypic features of each proband. The fourth column denotes hypothesis-driven RNA-seq analysis findings, while the fifth column displays the tissue used for RNA-seq and the results of the additional hypothesis-independent (HI) RNA-seq analysis, where applicable.

| **Case ID, Gene, Phenotype(s), Inheritance, Transcript** | **Variants** | **Primary Clinical Features** | **Hypothesis-driven RNA-seq Analysis [Outcome]** | | **Tissue used/ HI RNA-seq Analysis Outcomes (where relevant)** |
| --- | --- | --- | --- | --- | --- |
| **Putative splice variant outside canonical splice site** | | | | | |
| **Case 1**  ***SASS6***  OMIM: 616402 (AR) NM_194292.3 | c.39_49del (p.Val14Arg*3) [VUS, mat]   **c.207-11C>A (p.?) [VUS, pat]** | Male with microcephaly, abnormal brain MRI (abnormality of neuronal migration, abnormal corpus callosum and brain stem, hypoplastic left olfactory bulb and likely right persistent hypertrophic primary vitreous), seizures, GDD, exudative vitreoretinopathy. | The intronic variant leads to the leaky exon 4 skipping. This is observed in only ~30% of transcripts, which causes in-frame deletion of 35 amino acids, including 22 of the highly conserved PISA domain, essential for SASS-5 interaction **[Diagnostic]**. | | Tissue: LCLs  HI RNA-seq: N/A |
| **Case 2**  ***NFU1***  OMIM: 605711 (AR), 620938 (AR) NM_ 001002755.4 | **c.62+89G>A, (p.?) [VUS, mat]**   c.545G>A (p.Arg182Gln) [LP, pat] | Male with rapid neurologic deterioration, seizure, GDD, abnormal muscle tone, motor regression, acute encephalopathy, muscle weakness, upper motor neuron dysfunction, hyperreflexia, abnormality of the eye, type-2 muscle fiber atrophy, loss of consciousness, bradycardia, heart murmur, abnormal respiratory system physiology, respiratory tract infection, pleural effusion, fever, vomiting. | The LP missense variant alters the last nucleotide of exon 6, leading to out-of-frame skipping of exon 6.Only 5% of reads express the LP variant, suggesting a 95% skew towards the reference allele, likely due to NMD. The intronic variant creates a new deep intronic splice site at c.62+89G>A that extends exon 1 by ~90 nucleotides, replacing the original splice donor site. Notably, this novel donor site aligns with an alternate version of exon 1 in a less expressed transcript. Additionally, reduced coverage of exon 3 is seen, consistent with two other minimally expressed transcripts. Together, these findings suggest a switch to a less biologically relevant transcript, while maintaining the reading frame **[Diagnostic]**. | | Tissue: Fibroblasts  HI RNA-seq: N/A |
| **Case 3 *PPP1R2***  NM_006241.8 | **c.403+3A>T (p.?) [VUS, mat and pat]** | Female with intrauterine (IUGR) and postnatal growth restriction, ventricular septal defect, dysmorphic features (proptosis, long eyelashes, thick eyebrows, low-set ears), plagiocephaly, microcephaly, sensorineural hearing loss, cortical cataracts, abnormality of the retina, intellectual disability with delayed speech and language development, and autism spectrum disorder. | The homozygous splice donor variant nearly abolishes splicing at the canonical splice donor site of exon 4, causing exon 4 skipping. This results in significantly reduced expression of both exons 4 and 5, along with intron 4-6 retention in some reads. This variant likely triggers a high level of NMD, though a small number of normal transcripts are retained.**[RNA VUS;** diagnosis remains in question as this is a candidate gene with no disease association**]**. | | Tissue: Whole Blood  HI RNA-seq: N/A |
| **Case 4 *PAPSS2***  OMIM: 612847 (AR) NM_001015880.2 | **c.1222G>A (p.Asp408Asn) [VUS, pat]**   **c.1087-4951_1087-4 [VUS, mat]** | Male with IUGR, failure to thrive, developmental delay, myopia, dysmorphisms, metaphyseal dysplasia, and associated hypercalcemia, severe short stature, mild bilateral sensorineural hearing loss | The missense variant substitutes the last nucleotide of exon 10 and creates a novel donor site within exon 10. This is supported by only a few reads and did not reduce usage of the original donor site. No splicing changes seen from the deep intronic variant **[Diagnosis Ruled Out]**. | | Tissue: Whole Blood  HI RNA-seq: No candidates identified |
| **Case 5**  ***USP34***  NM_014709.4 | **c.132-3T>C [VUS, mat]**  c.9025C>T:p.Arg3009Cys [VUS, Pat] | Female with clinical osteogenesis imperfecta. IUGR, Wormian bone, flaring of rib cage, skeletal dysplasia, metaphyseal widening, fractures of the long bones, rhizomelia. | The intronic variant did not impact splicing or expression **[Diagnosis Ruled Out]**. | | Tissue: Fibroblasts  HI RNA-seq: No candidates identified |
| **Case 6 *COL11A1*** OMIM: 604841 (AD), 228520 (AR), 154780 (AD)  NM_001854.4 | **c.4186-68_4186-65dup [VUS, inheritance unknown]** | Male with skeletal dysplasia, abnormal gait, lower limb hypertonia and hyperreflexia. | The intronic duplication variant did not impact splicing or expression **[Diagnosis Ruled Out]**. | | Tissue: Fibroblasts  HI RNA-seq: No candidates identified |
| **Case 7 *NFASC*** OMIM: 618356 (AR)  NM_001005388.3 | **c.536-3T>A [VUS, pat]**   **c.3020-95G>A [VUS, mat]** | Male with septo-optic dysplasia phenotype. Panhypopituitarism (hypoglycemia, hypernatremia, growth hormone deficiency, overweight), GDD, optic nerve hypoplasia and seep disturbance. Nystagmus, hypotonia, depressed nasal bridge, high arched palate. | Neither the splice acceptor nor the intronic variant impacted splicing. However, the expression of the gene was slightly upregulated. **[Diagnosis Ruled Out]**. | | Tissue: Fibroblasts  HI RNA-seq: No candidates identified |
| **Case 8 *PIEZO1*** OMIM: 194380 (AD), 616843 (AR)  NM_001142864.4 | **c.2991+7C>T [VUS, inherited pat and mat]** | Male with non-immune hydrops and persistent bilateral pleural effusions considered to be chylothoraxes. | The homozygous splice donor variant creates a novel splice site within intron 21, extending exon 21 by 5 nucleotides. This exon extension is observed in all reads, leading to a frameshift that affects all transcripts **[Diagnostic]**. | | Tissue: Whole Blood  HI RNA-seq: N/A |
| **Case 9**  ***ELN***  OMIM: 123700 (AD), 185500 (AD)  NM_000501.4 | **c.1719 T>A, p.(V573=) [VUS, pat]** | Male with supravalvular aortic stenosis, branch pulmonary artery stenosis, and mild facial differences (high anterior hair line, long face, epicanthal folds, broad nasal bridge, somewhat bulbous nasal tip, long philtrum, large and fleshy ear lobes, full lips). No features of cutis laxa. Proband’s father had no known phenotype, while the proband’s brother with the variant is affected with supravalvular aortic stenosis, bilateral pulmonary artery stenosis, coarctation of the aorta and hypoplastic arch. | The synonymous variant had almost no detectable expression with multiple disrupted splice junctions near the variant site. This was supported by a strong allele skew (over 90%) toward the reference allele, likely due to NMD of transcripts with the variant **[Diagnostic]**. | | Tissue: Fibroblasts  HI RNA-seq: N/A |
| **Case 10 *FOXRED1*** OMIM: 618241 (AR)  NM_017547.4 | c.733+1G>A (exon 6) [P, Mat]   **c.536+5G>A (exon 4) [VUS, Pat]** | Male with severe microcephaly, neonatal seizures, exaggerated startle reflex, appendicular hypertonia, brisk reflexes, MRI brain showing cystic white matter lesions in the periventricular white matter, lissencephaly in the anterior frontal lobes, abnormal brainstem and corpus callosum, small size of cerebellum, bilateral IVH (grade 2) and ventriculomegaly, and possible periventricular heterotopias. Also had congenital CMV infection, elevated blood lactate, elevated CK. | Both splice donor variants in introns 4 and 6 contribute to the skipping of exons 5 and 6, as evidenced by reduced expression of these exons and intron retention in the surrounding region. This likely results in NMD of most transcripts **[Diagnostic]**. | | Tissue: Whole Blood  HI RNA-seq: N/A |
| **Case 11 *PQBP1***  OMIM:309500 (XLR) NM_ 001032382.2 | **c.292+5G>A [VUS, mat]** | Male with multiple syringomas, hyperpigmentation on the arms and legs, developmental delay, ADHD, intellectual disability, and distinct facial features (overall appearance is long, nose is not prominent, small ears, almond shaped eyes, Slight asymmetry R side slightly larger than left). | The splice donor variant creates a novel splice site in intron 4 that extends exon 4 by 12 nucleotides in 22% of transcripts. While this exon extension is likely in-frame, the presence of very low levels of intron retention suggests the potential for some NMD **[RNA VUS;** not diagnostic, uncertain if impact is deleterious enough to be disease-causing]. | | Tissue: Whole Blood  HI RNA-seq: No candidates identified |
| **Case 12 *PIEZO2*** OMIM: 114300 (AD), 108145 (AD), 617146(AR), 248700 (AD)  NM_022068.4 | **c.7743-8A>G [VUS, mat and pat]** | Female with suspected congenital myopathy, in the context of a history of infantile hypotonia, motor delay, neuromuscular scoliosis, OSA, bilateral clubfeet, ureteric/renal calculi, anxiety and depression. Abnormal EEG (supporting a dx of epilepsy) but has never experienced a seizure. | The homozygous splice acceptor variant creates a novel splice site in intron 53, extending exon 54 by 7 nucleotides. This exon extension is present in all reads, resulting in a frameshift that affects all transcripts. Consequently, expression of exons 53 & 54 is reduced by ~50%, with intron 53 retention seen in half of the transcripts **[Diagnostic]**. | | Tissue: LCLs  HI RNA-seq: N/A |
| **Case 23 *TARS2*** OMIM: 615918 (AR)  NM_025150.5 | **c.470C>T (p.Thr157Ile) [VUS, Pat]** c.1487C>T (p.Thr496Ile) [VUS, Mat] | Female with seizures, hypsarrhythmia, intractable epilepsy, oral motor dysfunction, Severe developmental encephalopathy, infantile spasms, dystonia | The c.470C>T.Thr157Ile missense variant causes exon 4 skipping in a small number of reads.  No splicing impacts was observed for the c.1487C>T variant, suggesting its disease mechanism does not involve transcript disruption **[Transcript Effect Ruled Out]**. | | Tissue: Whole Blood  HI RNA-seq: No candidates identified. |
| **Case 49 *FLNA***  OMIM:  311300(XLD), 304120(XLD), 305620(XLR), and others.  NM_001110556.2 | **c.622+6G>A [VUS, Mat]** | Male with microcephaly, hypertelorism, low-set ears, downslanted palpebral fissures, abnormal skull morphology, osteopenia,  premature birth, disproportionate short stature, rhizomelia, hip subluxation, and periostitis. No maternal phenotype. | No abnormal splicing observed. Allele specific expression not assessed due to hemizygosity **[Diagnosis Ruled Out]**. | | Tissue: Whole Blood  HI RNA-seq: No candidates identified. |
| **Case 50**  ***DPH1***  OMIM: 616901 (AR) NM_001383.6 | c.374T>C (p.Leu125Pro) (LP, pat**)**  **c.16A>G (p.Met6Val) (VUS, mat)** | Male with ventricular septal defect, cleft palate, epicanthus, eye defects, protruding ear, uplifted earlobe, bilateral single palmar creases, 5th finger clinodactyly, brachydactyly of both hands and feet, clinodactyly of the 5th finger, 2-3 toe syndactyly, global developmental delay, mild intellectual disability, autism, gait ataxia, migraine, arachnoid cyst, mild ventriculomegaly, and delayed myelination. | No impact on splicing was seen from the c.16A>G variant, suggesting its disease mechanism does not involve transcript disruption **[Transcript Effect Ruled Out]**. | | Tissue: Whole Blood  HI RNA-seq: No candidates identified. |
| **Case 51 *FBXL4*** OMIM: 615471 (AR)  NM_001278716.2 | c.1303C>T, p.Arg435* [P, pat]  **c.1703-4A>G [VUS, mat]** | Male with thin upper lip vermilion, macrocephaly, retrognathia, epicanthus, smooth philtrum, low-set ears, long eyelashes, pectus excavatum, soft, doughy skin, generalized hypotonia, plagiocephaly, failure to thrive, gastroesophageal reflux, hypoplasia of the corpus callosum, polymicrogyria, increased serum lactate, enlarged cisterna magna, heterotopia, inverted nipples, depressed nasal bridge, wide intermammillary distance, almond-shaped palpebral fissure, sparse hair, microtia, feeding difficulties, hyperintensity of cerebral white matter on MRI, and subdural hemorrhage. | The splice variant completely disrupts normal splicing, resulting in retention of intron 9 in the majority (~75-80%) of transcripts. While low-level intron retention is observed in controls, nearly all transcripts from the patient sample exhibited this retention. Additionally, significant allelic skew (80%) toward the allele with the splice variant suggests that the *in trans* allele with the stop gain variant is likely undergoing NMD **[Diagnostic]**. | | Tissue: Fibroblasts  HI RNA-seq: N/A |
| **Case 52 *ACADM*** OMIM: 201450 (AR)  NM_000016.6 | c.984del; p.Met328Ilefs*5 [P, mat**]**  **c.85C>A; p.Arg29= [VUS, pat]** | Female with clinical MCAD and additional features not explained by her metabolic condition including macrocephaly (97th %ile), obesity (weight, BMI > 99th %ile) with normal stature, acanthosis nigricans, borderline precocious puberty (menarche at age 9), and specific learning disability. | The synonymous variant significantly increased skipping of exon 2 in ~50% of reads, compared to minimal levels of exon 2 skipping seen in tissue-matched controls, resulting in a frameshift **[Diagnostic]**. | | Tissue: Whole Blood  HI RNA-seq: N/A |
| **Case 53**  ***UFC1***  OMIM: 618076 (AR)  NM_016406.4 | **c.333-14T>C p.? [VUS, Mat]**  c.435G>A p.Trp145* [VUS, Pat] | Male with coarse female features, laryngomalacia, strabismus, esotropia, seizure, global developmental delay, axial hypotonia, and infantile spasms. | In both the proband and maternal samples, the VUS causes a 10-fold increase in exon 5 skipping (300 reads vs 30 reads in tissue matched controls) , reducing exon 5 expression by 25%. The stop gain variant, located in the last exon, likely escapes NMD, resulting in a truncated protein product **[RNA VUS;** diagnosis remains uncertain, functional studies required to confirm significance.**]**. | | Tissue: Whole Blood  HI RNA-seq: N/A |
| **Canonical splice site variant (CSSV)** | | | | | |
| **Case 13**  ***EFTUD2***  OMIM: 610536 (AD) NM_004247.3 | **c.702+1del (p.?) [P, *de novo*]** | Female with tracheoesophageal fistula, unilateral anotia, scoliosis, sensorineural hearing loss, no history of DD/ID, normal head circumference. | The splice donor variant in intron 9 creates a novel splice acceptor site, shortening exon 10 by 1 nucleotide in 11/89 reads. This change leads to a frameshift, which is further supported by reduced expression of *EFTUD2* mRNA **[Diagnostic]**. | | Tissue: Whole Blood  HI RNA-seq: N/A |
| **Case 14**  ***TBX6***  OMIM:122600 (AD/AR) NM_004608.4 | **c.118+2T>C [P, pat]**   T-C-A haplotype (c.1227G>A, c.-48-240A>G, -49+34G>T) [risk allele, mat] | Male with L3 hemivertebra with congenital thoracolumbar scoliosis, pre-axial polydactyly, and situs inversus totalis with dextrocardia | The paternal splice donor variant in intron 2 causes skipping of the first coding exon, which contains the start site. Consequently, a new downstream start site is used, causes an N-terminal truncation in 80% of the proband’s transcripts, while only 30% of the father’s transcripts are affected. This difference likely reflects the exacerbated effect in the proband due to the permissive haplotype present in *in trans*. This finding is further supported by the proportionally reduced expression of exon 1 **[RNA VUS;** diagnosis remains uncertain, functional studies required to confirm significance**]**. | | Tissue: LCLs  HI RNA-seq: N/A |
| **Case 15**  ***MED14***  NM_004229.4 | **c.2365+2T>C [VUS, mat]** | Male with clinical VLCAD, microcephaly and brain anomalies | The splice donor variant in intron 18 creates a novel splice donor site in exon 18, detected in 1.7% of transcripts. This results in an out-of-frame loss of the terminal end of exon 18. [**RNA VUS**]. | | Tissue: LCLs  HI RNA-seq: No candidates identified. |
| **Case 16 *RABGAP1***  NM_012197.4 | **c.591-1G>T [VUS, mat and pat]** | Female with microcephaly, GDD, ID, sensorineural hearing loss, delayed myelination and a thin corpus callosum. | The homozygous splice acceptor variant in intron 4 creates a novel splice site that shortens exon 5 by 8 bp in most transcript, while the remaining transcripts demonstrate intron retention. No normal splicing was observed. This leads to a frameshift and introduction of a premature stop codon. **[RNA VUS;** diagnosis remains in question as this is a candidate gene with no disease association**]**. | | Tissue: LCLs  HI RNA-seq: N/A |
| **Case 17 *SYNGAP1***  OMIM: 612621 (AD)  NM_ 006772.3 | **c.1914-1G>C [P, inheritance unknown; not mat]** | Male with GDD, ID, epilepsy, and neurocognitive profile and aggressive outbursts potentially out of keeping with *SYNGAP1*-related intellectual disability. | The splice acceptor variant in intron 11 creates a novel splice variant within exon 12, resulting in a 13bp exon shortening in 20% of transcripts. This out-of-frame event is accompanied by low levels of intron retention and allelic skew (77%) toward the reference allele, suggesting that some transcripts containing the variant are subject to NMD **[Diagnostic]**. | | Tissue: LCLs  HI RNA-seq: N/A |
| **Case 18: Compound heterozygote for Putative splice variant and CNV** | | | | | |
| **Case 18 *TGM1***  OMIM: 242300 (AR) NM_000359.2 | **c.985-3C>G, [VUS, *de novo*]**   **~3.58 kb Duplication**  **Chr14:24253090-24256672 (GRCh38), [LP, Mat]** | Female with lamellar Ichthyosis. | The maternally inherited duplication of exons 10-14 was confirmed to be in-tandem and out-of-frame, with intron retention observed outside the duplication boundary. The *de novo* splice acceptor variant in intron 6, confirmed to be *in trans*, results in the out-of-frame skipping of exon 7 in half of transcripts **[Diagnostic]**. | | Tissue: Fibroblasts  HI RNA-seq: N/A |
| **Copy number variants** | | | | | |
| **Case 19 *COL4A5*** OMIM:301050 (XLD) | **~23kb duplication; exons 10-24**  **chrX:108,574,886-108,598,389 (GRCh38) [LP, mat]** | Male with thin glomerular basement membrane with mild hematuria and proteinuria. Family members (one sister, one brother, mother and maternal grandfather similarly mildly affected). | The exon 10-24 duplication was confirmed to be in tandem and in-frame **[Diagnostic]**. | | Tissue: Fibroblasts  HI RNA-seq: N/A |
| **Case 20 *CDKL5***  OMIM:300672 (XLD) | **105 kb duplication; exons 2-5**  **ChrX:18474185-18579246 (GRCh38) [LP, presumed *de novo*]** | Male with central hypotonia and infantile spasms. | The exon 2-5 duplication was confirmed to be in-frame and in tandem. However, some intron retention is seen, which likely triggers NMD **[Diagnostic]**. | | Tissue: LCLs  HI RNA-seq: N/A |
| **Case 21 *TONSL*** OMIM: 271510 (AR)  NM_013432.5 | c.1459G>A:p.Glu487Lys [P, Pat]   **467bp exonic duplication**  **Chr8: 144430363-144430830**  **(GRCh38) [VUS, mat]** | Female with severe short stature, multiple skeletal anomalies, neutropenia, persistence of hemoglobin F, hypothyroidism (resolved), dysmorphic features and cafe au lait macules. | The maternal exon 25 duplication was confirmed to be in tandem and out-of-frame, causing a frameshift that likely triggers NMD. No duplication seen in proband’s sample possibly due to loss of the variant in the cell culture. **[Diagnostic]**. | | Tissue: LCLs  HI RNA-seq: N/A |
| **Case 22 *FGF13***  OMIM:301058 (XLD/XLR), 301095 (XLR), others. | **101bp insertion**  **X:138711393 (GRCh38) [VUS, mat]** | Male with GDD, hypotonia, and cognitive impairment. The proband’s maternal half-brother died at 8 months and had infantile spasms/developmental delay (not tested for variant), maternal uncle has GDD, ID, and psychosis (has the variant), and mother has had psychiatric diagnoses but no seizures or known ID. | No reads seen in the area of the duplication's insertion within the 5'UTR. No differences in expression level or splicing in any reads **[Unsuccessful]**. | | Tissue: Fibroblasts  HI RNA-seq: Not done as maternal sample used. |
| **Regulatory variants** | | | | | |
| **Case 24**  ***IDUA***  OMIM: 607016 (AR)  NM_000203.5 | c.1205 G>A, p.Trp402Ter [P, pat]  **c.-87 T>C [VUS, mat]** | Male with constrictive median neuropathy, contractures, arthropathy, myotendinitis, course small bone medulla, and osteopenia. | The 5’UTR variant is only present in 3% of transcripts, indicating a 97% skew toward the reference pathogenic allele **[Diagnostic]**. | | Tissue: Fibroblasts  HI RNA-seq: N/A |
| **Case 25**  ***NSD2***  OMIM: 619695 (AD) | **~37 kb deletion**  **Chr4:1837528-1874574 (GRCh38), [VUS, *de novo*]** | Female with distinctive facial features (broad nasal bridge with tubular nose, deep set eyes and straight eyebrows), developmental delay with speech delay, feeding difficulties, hypotonia. No typical growth parameters described with NSD2-related disorder (proband has normal to large body size; normal head size). | The deletion includes non-coding exon 1 of *NSD2*, which forms part of the 5’UTR 2, as well as the last three exons of *LETM1*, Deletion did not lead to any changes in expression or splicing of either gene **[Diagnosis Ruled Out]**. | | Tissue: LCLs  HI RNA-seq: No candidates identified |
| **Case 26 *EOLA1*** NM_01171907.3 | **c.-30+8C>T [VUS, mat]** | Male with anal malformations, autism spectrum disorder, anemia, autoinflammation resulting in multiple positive autoantibodies, lymphadenopathy with hypergammaglobinaemia, high IL-6 levels and plasma cell infiltrates, poor weight gain, chronic cough and eczema. | The 5’UTR variant did not impact splicing or expression **[Diagnosis Ruled Out]**. | | Tissue: LCLs  HI RNA-seq: No candidates identified |
| **Case 27**  ***MBD5***  OMIM: 156200 (AD) | **~138kb duplication** **Chr2:148197271-148335082 (GRCh38) [VUS, mat]** | Female with GDD with marked language delay and suspected ID, mother had learning difficulties. | The duplication encompassing part of the 5’UTR of *MBD5* did not lead to any changes in expression or splicing **[Diagnosis Ruled Out]**. | | Tissue: LCLs  HI RNA-seq: No candidates identified |
| **Case 28**  ***MAMLD1***  OMIM: 300758 (XLR) | **~407 kb duplication**  **ChrX:149960910-150367537 (GRCh38) [VUS, mat]** | Male with left-sided congenital diaphragmatic hernia, hypospadias, query micropenis. | The duplication spans the 5’UTR of *MAMLD1* and includes the last three exons of the upstream *EOLA2* gene. Split reads are seen at the 3’ duplication breakpoint downstream of the *MAMLD1* 5’UTR, mapping to exons 7 and 8 of the *BEND2* gene, located at the other end of the chromosome (Xp22.13). This suggests a fusion event and partial expression of *BEND2*, which is absent in tissue-matched controls **[RNA VUS]**. | | Tissue: Fibroblasts  HI RNA-seq: Not done as maternal sample used. |
| **Probands with no eligible variants** | | | | | |
| **Case ID** | **Primary Clinical Features** | | | **Tissue used/ HI RNA-seq Analysis Outcomes** | |
| **29** | GDD, epilepsy and bilateral cataracts. | | | Tissue: LCLs  HI RNA-seq: No candidates identified | |
| **30** | Myoclonic epilepsy (progressive), GDD, ataxia, cerebellar atrophy. | | | Tissue: LCLs  HI RNA-seq: No candidates identified | |
| **31** | Male with dolichocephaly, prominent occiput, long philtrum, low-set ears, wide nasal bridge, broad nasal tip, telecanthus, upslanted palpebral fissure, eczema, petechiae, anemia, neutropenia, bruising susceptibility, urticaria, prominent fingertip pads, failure to thrive, IUGR, hypoplastic toenails, diarrhea, lymphadenopathy, clinodactyly of the 5th finger, underdeveloped supraorbital ridges, flat nasal alae, allergy, and ecchymosis. | | | Tissue: Blood  HI RNA-seq: No candidates identified | |
| **32** | Features of Muscular Dystrophy. | | | Tissue: Muscle  HI RNA-seq: No candidates identified | |
| **33** | Subacute proximal weakness, neuromuscular disorder. | | | Tissue: Muscle  HI RNA-seq: No candidates identified | |
| **34** | Obstructive sleep apnea, sleep disorder breathing, seizures, Immunodeficiency, congenital heart disease. Developmental disability, GDD, failure to thrive, feeding difficulty, short stature, large head. | | | Tissue: LCLs  HI RNA-seq: No candidates identified | |
| **35** | IUGR, truncal hypotonia and appendicular hypertonia and hyperreflexia, encephalopathy, (hyperinsulinemic) hypoglycemia, requirement of respiratory support | | | Tissue: Muscle  HI RNA-seq: No candidates identified | |
| **36** | Microcephaly, micrognathia, malar hypoplasia, borderline aortic root dilatation, significant distal muscle weakness with decreased muscle mass, camptodactyly of several digits on both hands, bilateral hallux valgus | | | Tissue: Blood  HI RNA-seq: No candidates identified | |
| **37** | Myoclonus dystonia | | | Tissue: Fibroblasts  HI RNA-seq: No candidates identified | |
| **38** | Axial hypotonia, appendicular hypertonia, dysmorphic features, Pierre Robin sequence, micrognathia. | | | Tissue: Fibroblasts  HI RNA-seq: No candidates identified | |
| **39** | SDC, mega epiphyseal dysplasia | | | Tissue: Fibroblasts  HI RNA-seq: No candidates identified | |
| **40** | Ketotic hypoglycemia, severe metabolic acidosis | | | Tissue: Fibroblasts  HI RNA-seq: No candidates identified | |
| **41** | Female with axial hypotonia, history of generalized tonic clonic seizures, dysmorphic features (microcephaly, arched brows, long eyelashes, bulging eyelids & ptosis bilaterally, epicanthus, exophthalmos and infra-orbital crease, stridor micrognathia, inverted wide-spaced nipples, high arched palate, upturned nares & narrowing of the midface), horizontal nystagmus, GDD, (inability to walk, delayed speech and language development), and recurrent UTIs; family history: brother with autism, born premature (24 weeks). | | | Tissue: Fibroblasts  HI RNA-seq: No candidates identified | |
| **42** | Male with bilateral microtia, micrognathia and submucous cleft palate. Family history is significant for two paternal aunts with congenital hearing loss and visual impairment | | | Tissue: LCLs  HI RNA-seq: No candidates identified | |
| **43** | Proband had additional diagnosis of Chromosome 3p26.1 deletion. Asthma, ASD, colitis eosinophilic, constipation, epidural cyst L4-L5, dysphagia, gastroesophageal reflux disease, hypotonia, premature adrenarche, scoliosis, syringohydromyelia Syringomyelia C4-conus, Von Willebrand disease, type \| | | | Tissue: Blood  HI RNA-seq: No candidates identified | |
| **44** | IUGR with postnatal growth delay (height and weight below the 0.1%), vision loss in right eye secondary to a hypoplastic optic nerve, GDD, ASD. | | | Tissue: Blood  HI RNA-seq: **Candidate identified;** out-of-frame leaky exon 67 skipping in *HUWE1*. **[RNA VUS**]. | |
| **45** | Male with suspected congenital myasthenic syndrome, favorable response to Mestinon and Salbutamol. Progressive fatigable proximal muscle weakness, scapular winging, exercise intolerance, myalgia, arthralgia and fatigue. Hypotonia, fine motor delay, torticollis, scoliosis. Episodic and fatigable ptosis, lower facial weakness. Developmental motor disorder, toe walking, spastic bladder. Sacral cleft sinus, dysmorphic features (rounded and low set ears with pursed lips), anxiety, Tourette syndrome. IgA deficiency, recurrent infections, chronic sinusitis, ear tubes, brachial cyst and tonsils removed. | | | Tissue: Muscle  HI RNA-seq: No candidates identified | |
| **46** | Male with microcephaly, GDD/ID, spastic CP, infantile spasms/refractory epilepsy, epileptic encephalopathy, spastic cerebral palsy, nystagmus, cortical visual impairment, hyperreflexia. Adenoidal hypertrophy, elevated liver enzymes. GERD, G-tube, recurrent aspiration pneumonia, asthma, eczema, OSA, respiratory failure. | | | Tissue: Blood  HI RNA-seq: No candidates identified | |
| **47** | Features reminiscent of Meier-Gorlin syndrome. ARM (anorectal malformation), craniosynostosis of multiple cranial sutures, left sensory esotropia, blepharophimosis syndrome, left amblyopia, eating disorder, short stature, vision loss, learning disability, restrictive eating disorder, right hip pain, delayed bone maturation and bifid thumb. | | | Tissue: LCLs  HI RNA-seq: No candidates identified | |
| **48** | Male teenage with epilepsy (absence seizures, generalized and, myoclonic seizures), hypoplasia of the corpus callosum, abnormal morphology of the hippocampus, developmental regression, anxiety, learning disability, attention deficit hyperactivity disorder, hypotelorism, thickened helices, small nail, clinodactyly of the 2nd toe, long toe and thrombocytopenia. | | | Tissue: Blood  HI RNA-seq: No candidates identified | |
